# Severe COVID-19 outcomes by cardiovascular risk profile in England in 2020: a population-based cohort study

**DOI:** 10.1016/j.lanepe.2023.100604

**Published:** 2023-03-07

**Authors:** Charlotte Warren-Gash, Jennifer A. Davidson, Helen Strongman, Emily Herrett, Liam Smeeth, Judith Breuer, Amitava Banerjee

**Affiliations:** a Department of Non-Communicable Disease Epidemiology, London School of Hygiene and Tropical Medicine, London, UK; b Department of Infection, Immunity and Inflammation, UCL Great Ormond Street Institute of Child Health, University College London, London, UK; c Institute of Health Informatics, University College London, London, UK

**Keywords:** Cardiovascular risk, QRISK3, Hypertension, Severe COVID-19 outcomes, Electronic health records, Cohort study

## Abstract

**Background:**

While cardiovascular disease (CVD) is a risk factor for severe COVID-19, the association between predicted cardiovascular risk and severe COVID-19 among people without diagnosed CVD is unclear.

**Methods:**

We carried out historical, population-based cohort studies among adults aged 40–84 years in England using linked data from the Clinical Practice Research Datalink. Individuals were categorized into: existing CVD, raised cardiovascular risk (defined using QRISK3 score ≥10%) and low risk (QRISK3 score <10%) at 12/03/2020. We described incidence and severe outcomes of COVID-19 (deaths, intensive care unit [ICU] admissions, hospitalisations, major adverse cardiovascular events [MACE]) for each group. Among those with a COVID-19 record to 31/12/2020, we re-classified cardiovascular risk at infection and assessed the risk of severe outcomes using multivariable Cox regression with complete case analysis. We repeated analyses using hypertension to define raised cardiovascular risk.

**Findings:**

Among 6,059,055 individuals, 741,913 (12.2%) had established CVD, 1,929,627 (31.8%) had a QRISK3 score ≥10% and 3,387,515 (55.9%) had a QRISK3 score <10%. Marked gradients were seen in the incidence of all severe COVID-19 outcomes by cardiovascular risk profile. Among those with COVID-19 (N = 146,760), there was a strong association between raised QRISK3 score and death: adjusted hazard ratio [aHR] 8.77 (7.62–10.10), N = 97,725, which remained present, though attenuated in age-stratified results. Risks of other outcomes were also higher among those with raised QRISK3 score: aHR 3.66 (3.18–4.21) for ICU admissions, 3.38 (3.22–3.56) for hospitalisations, 5.43 (4.44–6.64) for MACE. When raised cardiovascular risk was redefined by hypertension status, only the association with MACE remained: aHR 1.49 (1.20–1.85), N = 57,264.

**Interpretation:**

Individuals without pre-existing CVD but with raised cardiovascular risk (by QRISK3 score) were more likely to experience severe COVID-19 outcomes and should be prioritised for prevention and treatment. Addressing cardiovascular risk factors could improve COVID-19 outcomes.

**Funding:**

10.13039/501100011950BMA Foundation for Medical Research/10.13039/501100000833Rosetrees Trust, 10.13039/100004440Wellcome, 10.13039/501100000274BHF.

**Research in context**

**Evidence before this study**

While severe outcomes of COVID-19 occur more frequently among individuals with pre-existing health conditions, the role of underlying cardiovascular risk is incompletely understood. We searched PubMed from inception to 11 April 2022 using the terms ((“cardiovascular risk” OR “hypertension”) AND (“COVID-19” OR “SARS-CoV-2”) AND (“severe outcomes” OR “mortality”)). Due to a large number of results, we limited to studies of adults in non-specialist populations and filtered by systematic reviews and meta-analyses. Results were obtained from 28 relevant systematic reviews, many covering overlapping studies. Most included studies were small (100s–1000s of patients) and conducted among hospitalised COVID-19 patients. Cardiovascular disease, and to a lesser extent hypertension, were typically associated with raised risks of severe outcomes and death from COVID-19 in these studies. Later population-based studies show that existing cardiovascular disease and some individual cardiovascular risk factors (diabetes, hypertension) were associated with COVID-19-related deaths. No studies assessed cardiovascular risk using risk prediction tools such as QRISK3 which combine different elements of risk.

**Added value of this study**

This is, to our knowledge, the only study of COVID-19 outcomes to date to characterize underlying cardiovascular risk profile comprehensively using a validated risk prediction score (QRISK3) in a large population-based cohort. We demonstrate a gradient in the risks of hospitalisation, intensive care unit (ICU) admission, major adverse cardiovascular events (MACE) and mortality by cardiovascular risk level, with the highest incidence of severe outcomes occurring among individuals with existing cardiovascular disease, followed by those at raised cardiovascular risk then those at low risk. In cohorts with confirmed and suspected COVID-19, we show that having an elevated QRISK3 score was associated with a higher risk of all categories of severe outcomes after accounting for sociodemographic, lifestyle and clinical confounders, while hypertension status was associated only with a higher risk of MACE.

**Implications of all the available evidence**

Being at raised cardiovascular risk, defined by having an elevated QRISK3 score, is associated with severe outcomes after COVID-19. Individuals at raised cardiovascular risk represent an important target for COVID-19 prevention and management, as an addition to the current focus on those with diagnosed cardiovascular disease. Strategies to improve cardiovascular health could also improve outcomes following COVID-19.

## Introduction

By the end of 2020, the COVID-19 pandemic had led to an estimated 3 million deaths worldwide.^1^ Large, population-based studies show that existing cardiovascular disease (CVD) and some individual cardiovascular risk factors (such as diabetes and hypertension) are associated with COVID-19-related deaths.^2^, ^3^, ^4^ Other studies support associations between CVD or individual risk factors and severe COVID-19 outcomes among hospitalised patients.^5^, ^6^, ^7^ CVD is a component of the QCOVID risk prediction tool which predicts risks of hospitalisation and mortality from COVID-19.^8^ However, it is unclear how being at raised cardiovascular risk, defined by commonly-used clinical risk prediction tools such as QRISK3, affects severe COVID-19 outcomes among individuals without existing CVD. Such individuals were not considered ‘clinically vulnerable’ in England during the COVID-19 pandemic.^9^

Cardiovascular complications of COVID-19 have increasingly been recognized: population-based self-controlled case series studies from Scotland,^10^ Sweden^11^ and Denmark^12^ show an early elevation in acute cardiovascular events such as myocardial infarction (MI) and stroke following COVID-19. Similar transient elevations in the risks of MI and stroke occur following other laboratory-confirmed respiratory infections including influenza and *Streptococcus pneumoniae*.^13^ Although evidence from before the COVID-19 pandemic showed that such complications are more frequent after respiratory infections among individuals at raised cardiovascular risk,^14^ this has not been comprehensively investigated for COVID-19.

Population-based studies with detailed cardiovascular risk assessments are needed to assess the burden of acute severe outcomes of COVID-19, including cardiovascular complications, among individuals with differing levels of underlying cardiovascular risk to guide accurate stratified prevention and management. Here we aimed to quantify the incidence and severe outcomes of SARS-CoV-2 infections and to assess the risk of severe COVID-19 outcomes following infection by underlying cardiovascular risk profile among adults in England.

## Methods

### Data sources

We used the Clinical Practice Research Datalink (CPRD) Aurum^15^ January 2022 dataset, with individual level linked data from Hospital Episode Statistics Admitted Patient Care (HES APC), Office for National Statistics (ONS) deaths, Second Generation Surveillance System (SGSS) SARS-CoV-2, and COVID-19 Hospitalisations in England Surveillance System (CHESS).^16^

The CPRD Independent Scientific Advisory Committee (application 20_000135) and the London School of Hygiene and Tropical Medicine (LSHTM) Ethics Committee (application 22717) approved the study. CPRD provided relevant HES, ONS, SGSS and CHESS data for the study population. All code lists are published on LSHTM Data Compass.^17^

### Incidence study population and follow-up

**Fig. 1:**
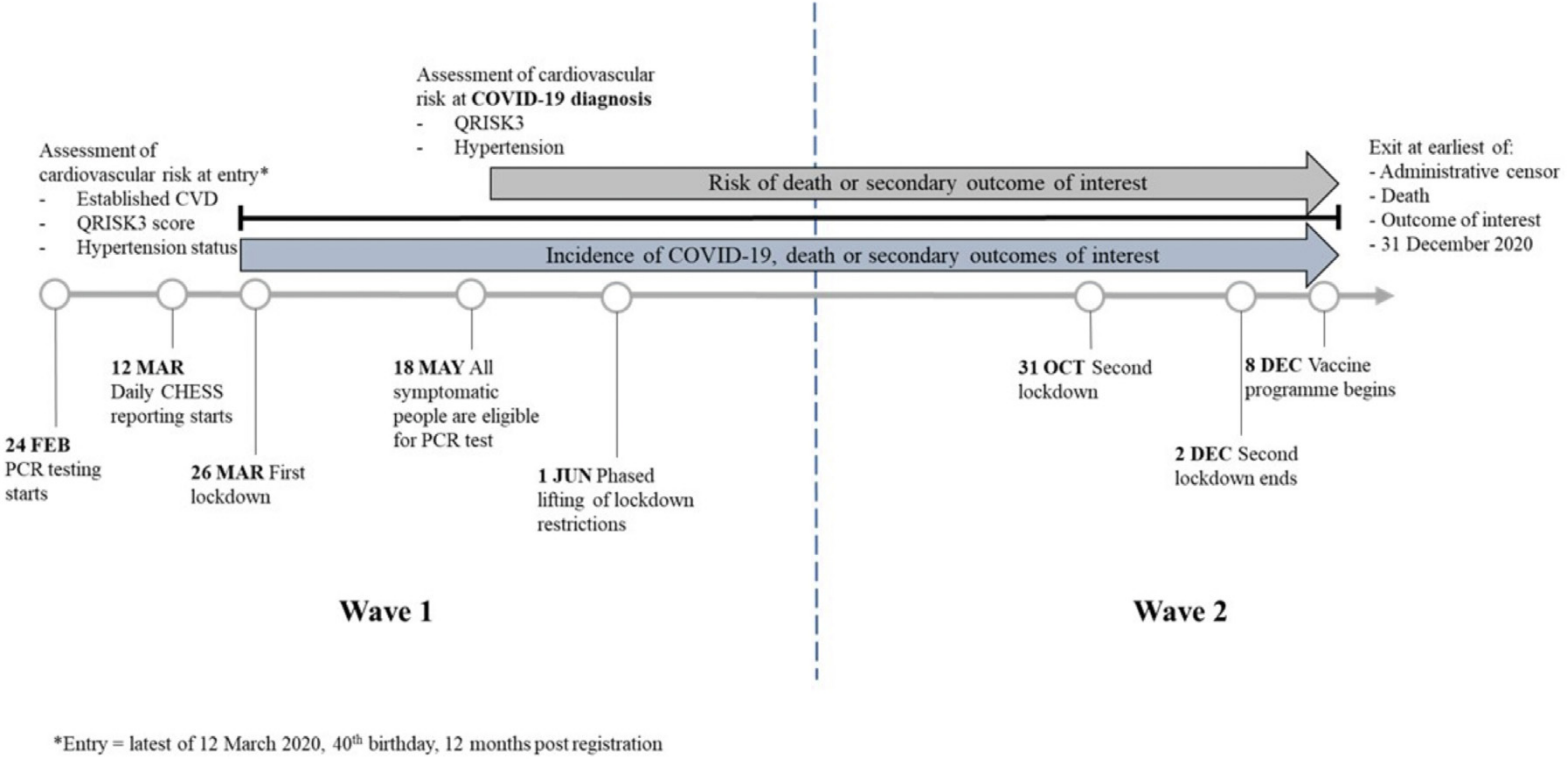
**Study design overview with 2020 England COVID-19 timeline**.

All individuals aged 40–84 years with at least one year of post-registration time at their primary care practice who are eligible for linkage to HES were eligible for inclusion in our incidence study. Follow-up of individuals started at the latest of; age 40 years, 12 months post-registration, or 12 March 2020, and ended at the earliest of; date of death or outcome of interest, administrative censor (date of leaving the practice or date of last data collection from the practice), or 31 December 2020 (Fig. 1). We started follow-up from 12 March 2020 when daily reporting to CHESS was initiated.^18^

### Cohort study population and follow up

Our cohort study included individuals with COVID-19. In our main analysis we defined this as laboratory-confirmed SARS-CoV-2, identified using SGSS and CHESS data. All individuals in either of the two datasets were considered to have SARS-CoV-2, with the date of infection taken as the earliest specimen date. In a secondary analysis, we defined COVID as clinically reported COVID-19 (CPRD or HES APC [any diagnostic position] recorded) without laboratory-confirmed SARS-CoV-2. We only considered one infection, the earliest recorded, per individual. Follow-up in the cohort study started at this date and ended at the earliest of the dates set out in our incidence study (Fig. 1). We stratified the study population further in time based on the UK COVID-19 waves (one; 12 March to 16 August and two; 17 August to 31 December), during which different testing practices were in operation.

### Outcomes

Our primary outcome of interest was death attributable to COVID-19. We defined COVID-19 attributable deaths as those coded as U07.1 or U07.2 in ONS data. In a sensitivity analysis we explored broadening our primary outcome of death attributable to COVID-19 to all-cause death which occurred within 28 days of the individual's diagnosis (based on test result among those with laboratory-confirmed SARS-CoV-2 or consultation date for those with clinically reported COVID-19). Our secondary outcomes were hospitalisation due to COVID-19 (defined by COVID-19 in the primary diagnosis field of any episode recorded in HES APC or presence in CHESS dataset), ICU admission due to COVID-19 (defined by ICU admission recorded in CHESS), need for respiratory support due to COVID-19 (defined by mechanical ventilation recorded in CHESS), or major adverse cardiovascular event (MACE [composite of acute coronary syndrome which included myocardial infarction and unstable angina, ischaemic stroke, acute left ventricular failure, or major ventricular arrhythmia recorded in CPRD or HES APC]). These definitions were informed by a systematic review of the validity of cardiovascular event recording in electronic health records.^19^

### Exposure

Our exposure of interest was cardiovascular risk. First, we identified individuals with established CVD (CPRD Aurum or HES APC recorded) diagnosed before baseline. Among individuals without CVD, we then used QRISK3 score to identify individuals with and without raised cardiovascular risk. Individuals with established CVD were included in our incidence study but excluded from our cohort study.

QRISK3 is a validated UK ten-year risk prediction score for myocardial infarction or stroke based on a combination of known risk factors,^20^ such as age, sex, ethnicity, socio-economic status, family history of coronary heart disease in a first degree relative aged <60 years, and comorbid health conditions (further outlined in Supplementary methods). We classified individuals as having raised cardiovascular risk (QRISK3 ≥10%) or low cardiovascular risk (QRISK3 <10%) at baseline, based on NICE thresholds for recommending statins for primary prevention.^21^ In a secondary analysis, we further stratified QRISK3 scores into <10%, 10–20%, or ≥20%.

In separate analyses, we redefined raised cardiovascular risk based on hypertension status within the five years before baseline as a pragmatic method to identify individuals at raised cardiovascular risk in settings where QRISK3 is not widely used. We classified hypertension using coded CPRD diagnoses or the most recent to baseline blood pressure (BP) reading with systolic BP of ≥140mmHg or diastolic BP of ≥90mmHg.

### Covariates

Covariates differed depending on the method used to define cardiovascular risk. In analyses where raised cardiovascular risk was defined by QRISK3, we included covariates which were not part of determining the QRISK3 score as detailed in the Supplementary methods. In analyses where hypertension was used to define raised cardiovascular risk, we included covariates accounted for in the QRISK3 algorithm as well as those not included in the algorithm and adjusted for in QRISK3 analysis, as outlined in the Supplementary methods.

### Statistical analysis

We described the baseline characteristics, for both the incidence and cohort study populations, using numbers and percentage for categorical variables and mean with standard deviation or median with interquartile range for continuous variables.

For our incidence study population, stratified by cardiovascular risk, we calculated incidence of the primary outcome of COVID-19 death and secondary outcomes of ICU admission, respiratory support, hospitalisation, and MACE, among the whole population, regardless of COVID-19 status. We then calculated the incidence of SARS-CoV-2 infection and clinically reported COVID-19, as well as our primary and secondary outcomes following laboratory-confirmed SARS-CoV-2 or clinically reported COVID-19. We further stratified results by time according to COVID-19 wave. Additionally, we generated age standardised incidence rates, stratified by sex, using one-year age bands from the ONS mid-year population estimates for 2020.^22^

Among our cohort study population (those with COVID-19), we used Cox proportional hazards regression finely adjusted for calendar time to generate hazard ratios for the association between cardiovascular risk and each outcome, initially adjusting models in hypertension analysis for age and sex, and then in a full model adjusted for all potential confounders. A complete case-analysis approach was used for multivariable analyses. We reported numbers in unadjusted and full models, compared characteristics of those included and excluded from the complete case analysis and also re-ran unadjusted models in the complete case analysis population. We did not conduct multiple imputation because data in CPRD are unlikely to be missing at random. We examined non-proportionality using Schoenfeld's residuals. In a post-hoc analysis, we stratified QRISK3 results by age group to evaluate the effect of age. There were no individuals aged 75–84 years with a QRISK3 score <10%, so age-stratified results were only generated for age groups of 40–54, 55–64, and 65–74 years. Age, like all risk factors included in the calculation of QRISK3 score, had not been adjusted for in the main QRISK3 analysis as the variable is also considered in the assignment of individual scores. However, in a further post-hoc analysis we additionally adjusted for age given the strong association between age and risk of severe COVID-19 outcomes.^2^ We conducted all analyses in Stata, version 16.

### Role of the funding source

The funders of the study had no role in study design, data collection, data analysis, data interpretation or writing of the report.

## Results

### Description of incidence study population

**Fig. 2:**
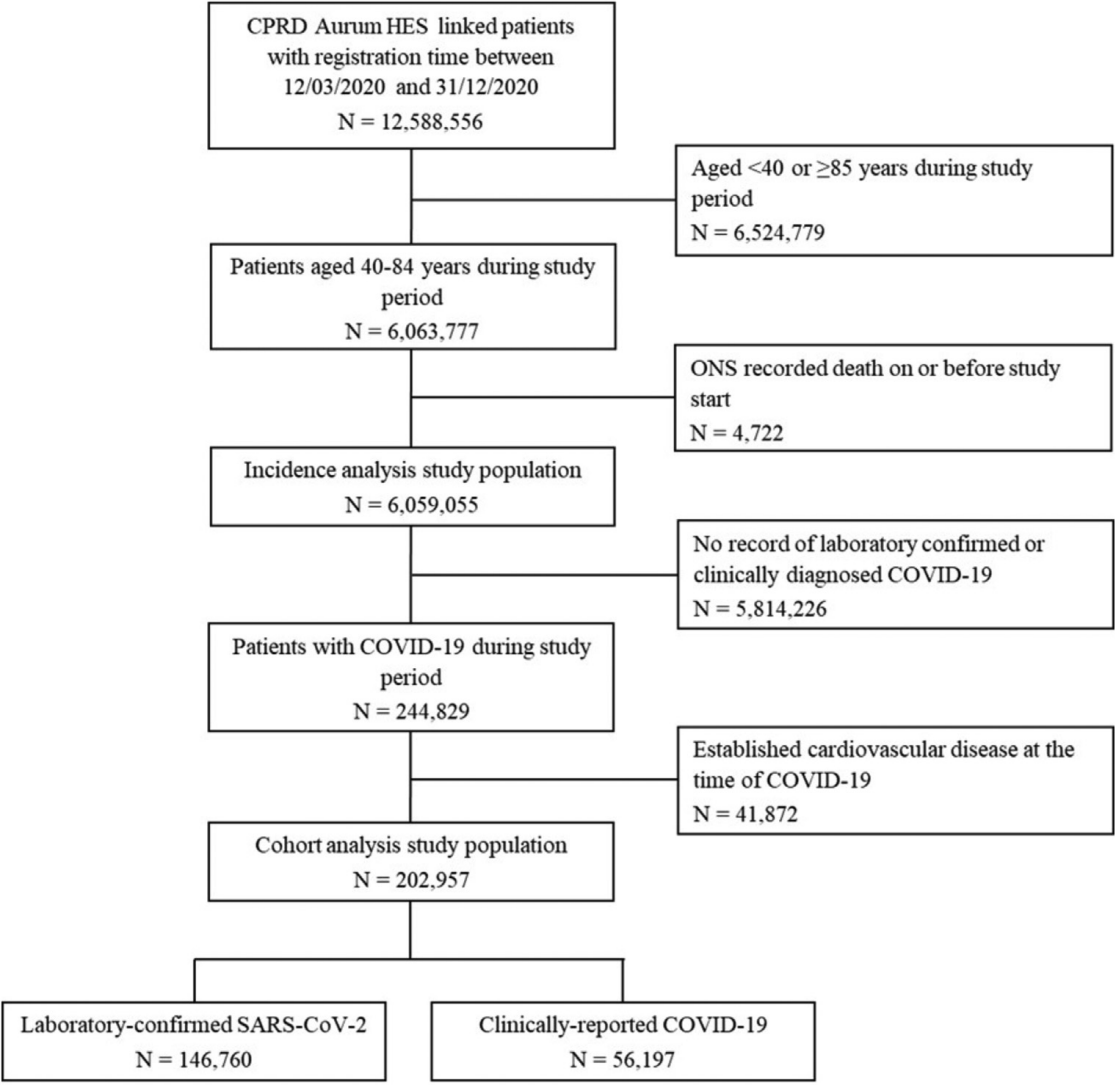
**Study population flow chart**.

The incidence study population included 6,059,055 individuals aged 40–84 years of age (Fig. 2), 12.2% (741,913) had established CVD and among those without established CVD, 31.9% (1,929,627) had a QRISK3 score ≥10% and 55.9% (3,387,515) had a QRISK3 score <10%, and 31.1% (1,881,654) had hypertension and 56.7% (3,435,488) had no hypertension. The baseline characteristics of the study population are described in Supplementary Table S1.

### Incidence of COVID-19 and severe outcomes

Among all individuals the incidence of COVID-19 death was 1.7 (95% CI 1.7–1.8) per 1000 with the highest incidence among those with established CVD (7.4 [7.2–7.7] per 1000), followed by those with raised cardiovascular risk (QRISK3 ≥10%; 2.2 [2.1–2.2] and hypertension; 1.4 [1.3–1.5] per 1000), and was lowest among those at low cardiovascular risk (QRISK3 <10%; 0.2 [0.2–0.2] and no hypertension; 0.7 [0.6–0.7] per 1000). The same gradient by cardiovascular risk level was observed for hospitalisations and MACE, and for outcomes among individuals with laboratory-confirmed SARS-CoV-2 and clinically reported COVID-19 (Table 1). Results by COVID-19 wave showed a higher incidence of outcomes of interest in wave 1 compared to the beginning of wave 2 (Supplementary Tables S2 and S3). Employing the sensitivity analysis definition of death (all cause within 28 days of diagnosis), resulted in similar incidence as COVID-19 death among individuals with laboratory-confirmed SARS-CoV-2 and a higher incidence than COVID-19 death among those with clinically reported COVID-19 (Table 1). Age standardized rates, stratified by sex, are shown in Supplementary Table S4. While age standardized rates showed similar trends across the cardiovascular risk levels, these rates were diminished compared to crude estimates.

**Table 1:** Number and incidence rates of laboratory-confirmed SARS-CoV-2 and clinically reported COVID-19 and outcomes of interest.

	All		Established CVD		QRISK3 score				Hypertension			
					Raised risk		Low risk		Raised risk		Low risk	
	N	Rate (95% CI) per 1000	N	Rate (95% CI) per 1000	N	Rate (95% CI) per 1000	N	Rate (95% CI) per 1000	N	Rate (95% CI) per 1000	N	Rate (95% CI) per 1000
All individuals	6,059,055		741,913		1,929,627		3,387,515		1,881,654		3,435,488	
COVID-19 death^a^	7866	1.7 (1.7–1.8)	4164	7.4 (7.2–7.7)	3203	2.2 (2.1–2.2)	499	0.2 (0.2–0.2)	2014	1.4 (1.3–1.5)	1688	0.7 (0.6–0.7)
Hospitalisation^b^	28,013	6.1 (6.0–6.2)	10,880	19.4 (19.0–19.8)	10,794	7.3 (7.2–7.4)	6339	2.5 (2.4–2.6)	8481	5.9 (5.8–6.0)	8652	3.4 (3.3–3.4)
Major adverse cardiovascular event	71,035	15.5 (15.4–15.6)	49,318	88.0 (87.2–88.8)	16,604	11.2 (11.0–11.4)	5113	2.0 (2.0–2.1)	12,633	8.8 (8.6–8.9)	9084	3.5 (3.4–3.6)

Laboratory-confirmed SARS-CoV-2	174,129	38.0 (37.8–38.2)	24,779	44.2 (43.7–44.8)	41,416	28.0 (27.7–28.2)	107,934	42.5 (42.2–42.7)	50,854	35.3 (35.0–35.6)	98,496	38.2 (37.9–38.4)
COVID-19 death^a^	6475	48.9 (47.7–50.1)	3493	199.9 (193.4–206.6)	2597	82.9 (79.8–86.2)	385	4.6 (4.2–5.1)	1664	42.5 (40.5–44.6)	1318	17.4 (16.5–18.4)
All cause death within 28 days of diagnosis	6649	50.2 (49.0–51.4)	3592	205.6 (198.9–212.4)	2661	85.0 (81.8–88.3)	396	4.7 (4.3–5.2)	1716	43.9 (41.8–46.0)	1341	17.7 (16.8–18.7)
ICU admission^c^	2024	15.3 (14.6–16.0)	499	28.6 (26.2–31.2)	930	29.7 (27.8–31.7)	595	7.1 (6.6–7.7)	811	20.7 (19.4–22.2)	714	9.4 (8.8–10.1)
Respiratory support^d^	1084	8.2 (7.7–8.7)	230	13.2 (11.6–15.0)	526	16.8 (15.4–18.3)	328	3.9 (3.5–4.4)	475	12.1 (11.1–13.3)	379	5.0 (4.5–5.5)
Hospitalisation^b^	17,893	135.2 (133.2–137.2)	6555	375.1 (366.1–384.3)	7047	225.0 (219.8–230.3)	4291	51.3 (49.8–52.9)	5628	143.9 (140.1–147.7)	5710	75.3 (73.4–77.3)
Major adverse cardiovascular event	2251	17.0 (16.3–17.7)	1422	81.4 (77.3–85.7)	616	19.7 (18.2–21.3)	213	2.5 (2.2–2.9)	486	12.4 (11.4–13.6)	343	4.5 (4.1–5.0)

Clinically reported COVID-19	70,700	15.4 (15.3–15.5)	13,668	24.4 (24.0–24.8)	20,240	13.7 (13.5–13.9)	36,792	14.5 (14.3–14.6)	21,374	14.8 (14.6–15.0)	35,658	13.8 (13.7–14.0)
COVID-19 death^a^	723	13.4 (12.5–14.4)	365	36.2 (32.6–40.1)	318	20.7 (18.5–23.1)	40	1.4 (1.0–1.9)	178	10.8 (9.3–12.5)	180	6.6 (5.7–7.6)
All cause death within 28 days of diagnosis	1599	29.6 (28.2–31.1)	823	81.5 (76.2–87.3)	657	42.7 (39.5–46.1)	119	4.2 (3.5–5.0)	375	22.8 (20.6–25.2)	401	14.6 (13.3–16.1)
Hospitalisation^b^	3692	68.4 (66.2–70.6)	1336	132.4 (125.5–139.7)	1385	89.9 (85.3–94.8)	971	34.1 (32.0–36.3)	1124	68.2 (64.3–72.3)	1232	44.9 (42.5–47.5)
Major adverse cardiovascular event	2002	37.1 (35.5–38.7)	1282	127.0 (120.3–134.2)	569	37.0 (34.0–40.1)	151	5.3 (4.5–6.2)	418	25.4 (23.0–27.9)	302	11.0 (9.8–12.3)

a Ascertained from ONS death certificate data in which the COVID related ICD-10 codes U07.1 or U07.2 were present in the record.

b Ascertained from presence in CHESS dataset or HES APC record coded with primary diagnosis of U07.1 or U07.2.

c Ascertained from CHESS records coded with ICU/HDU admission, only available for those with laboratory confirmed SARS-CoV-2.

d Ascertained from CHESS record coded with use of respiratory support via invasive mechanical ventilation, only available for those with laboratory confirmed SARS-CoV-2.

### Description of cohort study population

After excluding those with established CVD, 146,760 people had laboratory-confirmed SARS-CoV-2 and 56,197 had clinically-reported COVID-19 during our study period (Fig. 2). Among those with laboratory-confirmed SARS-CoV-2, when cardiovascular risk was classified by QRISK3 score, 26.8% (39,295) had raised risk (a score ≥10%) and 73.2% (107,465) had low risk (a score <10%). When hypertension was used to classify cardiovascular risk, 34.0% (49,955) had raised risk (hypertension) and 66.0% (96,805) had low risk (no hypertension). Individuals with laboratory-confirmed SARS-CoV-2 and raised cardiovascular risk (QRISK3 ≥10% or hypertension) were older and a higher proportion were men. Baseline characteristics of the laboratory-confirmed SARS-CoV-2 study population are shown in Table 2 and of the clinically reported COVID-19 study population in Supplementary Table S5. When compared to individuals with laboratory-confirmed SARS-CoV-2, a higher proportion of those with clinically reported COVID-19 were older, women, less affluent, and lived in London.

**Table 2:** Baseline characteristics of the laboratory-confirmed SARS-CoV-2 study population by cardiovascular risk.

	All	QRISK3 score		Hypertension	
		Raised risk	Low risk	Raised risk	Low risk
	N = 146,760	N = 39,295	N = 107,465	N = 49,955	N = 96,805
Age (years), Mean (SD)^a^	54.0 (10.1)	65.3 (9.5)	49.9 (6.6)	57.7 (10.6)	52.2 (9.3)
Age group (years)^a^					
40-54	84,928 (57.9%)	5150 (13.1%)	79,778 (74.2%)	21,382 (42.8%)	63,546 (65.6%)
55-64	39,757 (27.1%)	13,967 (35.5%)	25,790 (24.0%)	16,435 (32.9%)	23,322 (24.1%)
65-74	14,782 (10.1%)	12,885 (32.8%)	1897 (1.8%)	7819 (15.7%)	6963 (7.2%)
75-84	7293 (5.0%)	7293 (18.6%)	0 (0.0%)	4319 (8.6%)	2974 (3.1%)
Sex^a^					
Women	80,805 (55.1%)	14,316 (36.4%)	66,489 (61.9%)	24,608 (49.3%)	56,197 (58.1%)
Men	65,955 (44.9%)	24,979 (63.6%)	40,976 (38.1%)	25,347 (50.7%)	40,608 (41.9%)
Ethnicity^a^					
White or not stated	104,902 (71.5%)	28,657 (72.9%)	76,245 (70.9%)	36,280 (72.6%)	68,622 (70.9%)
South Asian	12,340 (8.4%)	4588 (11.7%)	7752 (7.2%)	3893 (7.8%)	8447 (8.7%)
Black	3408 (2.3%)	519 (1.3%)	2889 (2.7%)	1378 (2.8%)	2030 (2.1%)
Mixed/Other	11,793 (8.0%)	2624 (6.7%)	9169 (8.5%)	4114 (8.2%)	7679 (7.9%)
Unknown	14,317 (9.8%)	2907 (7.4%)	11,410 (10.6%)	4290 (8.6%)	10,027 (10.4%)
Townsend quintile^a^					
1 (most affluent)	28,068 (19.1%)	6224 (15.8%)	21,844 (20.3%)	9175 (18.4%)	18,893 (19.5%)
2	28,488 (19.4%)	6968 (17.7%)	21,520 (20.0%)	9619 (19.3%)	18,869 (19.5%)
3	28,259 (19.3%)	7281 (18.5%)	20,978 (19.5%)	9612 (19.2%)	18,647 (19.3%)
4	28,947 (19.7%)	8099 (20.6%)	20,848 (19.4%)	10,027 (20.1%)	18,920 (19.5%)
5 (least affluent)	32,940 (22.4%)	10,712 (27.3%)	22,228 (20.7%)	11,505 (23.0%)	21,435 (22.1%)
Unknown	58 (0.0%)	11 (0.0%)	47 (0.0%)	17 (0.0%)	41 (0.0%)
Region of residence					
North East	6207 (4.2%)	1746 (4.4%)	4461 (4.2%)	2276 (4.6%)	3931 (4.1%)
North West	34,059 (23.2%)	9696 (24.7%)	24,363 (22.7%)	12,364 (24.8%)	21,695 (22.4%)
Yorkshire and the Humber	4908 (3.3%)	1328 (3.4%)	3580 (3.3%)	1718 (3.4%)	3190 (3.3%)
East Midlands	2508 (1.7%)	690 (1.8%)	1818 (1.7%)	897 (1.8%)	1611 (1.7%)
West Midlands	24,071 (16.4%)	6916 (17.6%)	17,155 (16.0%)	8972 (18.0%)	15,099 (15.6%)
East of England	5347 (3.6%)	1207 (3.1%)	4140 (3.9%)	1662 (3.3%)	3685 (3.8%)
South West	32,542 (22.2%)	8765 (22.3%)	23,777 (22.1%)	10,067 (20.2%)	22,475 (23.2%)
South Central	26,156 (17.8%)	6050 (15.4%)	20,106 (18.7%)	8170 (16.4%)	17,986 (18.6%)
London	10,734 (7.3%)	2812 (7.2%)	7922 (7.4%)	3737 (7.5%)	6997 (7.2%)
Unknown	228 (0.2%)	85 (0.2%)	143 (0.1%)	92 (0.2%)	136 (0.1%)
BMI category^a^^,^^b^					
Underweight (<18.5 kg/m^2^)	865 (0.6%)	336 (0.9%)	529 (0.5%)	182 (0.4%)	683 (0.7%)
Normal (18.5–24.9 kg/m^2^)	25,789 (17.6%)	5980 (15.2%)	19,809 (18.4%)	5727 (11.5%)	20,062 (20.7%)
Overweight (25.0–29.9 kg/m^2^)	39,501 (26.9%)	12,415 (31.6%)	27,086 (25.2%)	13,618 (27.3%)	25,883 (26.7%)
Obese (30.0–39.9 kg/m^2^)	34,394 (23.4%)	12,652 (32.2%)	21,742 (20.2%)	16,361 (32.8%)	18,033 (18.6%)
Severely obese (≥40.0 kg/m^2^)	5558 (3.8%)	1985 (5.1%)	3573 (3.3%)	3153 (6.3%)	2405 (2.5%)
Unknown	40,653 (27.7%)	5927 (15.1%)	34,726 (32.3%)	10,914 (21.8%)	29,739 (30.7%)
Cholesterol:HDL, Mean (SD)^a^^,^^b^	3.8 (1.2)	4.0 (1.3)	3.7 (1.1)	3.8 (1.2)	3.7 (1.2)
Systolic blood pressure, Mean (SD)^a^^,^^b^^,^^c^	128.1 (14.6)	134.3 (14.4)	125.5 (13.8)	138.7 (13.5)	122.1 (11.3)
Smoking status^a^^,^^b^					
Non-smoker	73,789 (50.3%)	18,643 (47.4%)	55,146 (51.3%)	26,210 (52.5%)	47,579 (49.1%)
Ex-smoker	34,028 (23.2%)	12,543 (31.9%)	21,485 (20.0%)	13,287 (26.6%)	20,741 (21.4%)
Current smoker	12,400 (8.4%)	4630 (11.8%)	7770 (7.2%)	3872 (7.8%)	8528 (8.8%)
Unknown	26,543 (18.1%)	3479 (8.9%)	23,064 (21.5%)	6586 (13.2%)	19,957 (20.6%)
Alcohol consumption^b^					
No heavy drinking	85,406 (58.2%)	26,746 (68.1%)	58,660 (54.6%)	32,332 (64.7%)	53,074 (54.8%)
Heavy drinking	12,319 (8.4%)	3760 (9.6%)	8559 (8.0%)	4512 (9.0%)	7807 (8.1%)
Unknown	49,035 (33.4%)	8789 (22.4%)	40,246 (37.5%)	13,111 (26.2%)	35,924 (37.1%)
Family history of CHD^a^	13,116 (8.9%)	4579 (11.7%)	8537 (7.9%)	4371 (8.7%)	8745 (9.0%)
Consultation frequency in prior 12 months, Median (IQR)	3 (1–7)	6 (2–10)	3 (1–6)	4 (1–9)	3 (1–6)
Medication use^d^					
Regular corticosteroids^a^	1545 (1.1%)	1070 (2.7%)	475 (0.4%)	790 (1.6%)	755 (0.8%)
Antihypertensives^a^	35,232 (24.0%)	15,938 (40.6%)	19,294 (18.0%)	21,361 (42.8%)	13,871 (14.3%)
Statins	19,723 (13.4%)	13,931 (35.5%)	5792 (5.4%)	11,230 (22.5%)	8493 (8.8%)
Antiplatelets	7415 (5.1%)	4360 (11.1%)	3055 (2.8%)	3830 (7.7%)	3585 (3.7%)
Anticoagulants	2720 (1.9%)	1761 (4.5%)	959 (0.9%)	1359 (2.7%)	1361 (1.4%)
Comorbid condition					
Atrial fibrillation^a^	1420 (1.0%)	1303 (3.3%)	117 (0.1%)	745 (1.5%)	675 (0.7%)
Migraines^a^	5396 (3.7%)	907 (2.3%)	4489 (4.2%)	1559 (3.1%)	3837 (4.0%)
Diabetes^a^	12,238 (8.3%)	9811 (25.0%)	2427 (2.3%)	6603 (13.2%)	5635 (5.8%)
CKD stage 3–5^a^	9294 (6.3%)	7013 (17.9%)	2281 (2.1%)	5482 (11.0%)	3812 (3.9%)
Chronic liver disease	1563 (1.1%)	775 (2.0%)	788 (0.7%)	641 (1.3%)	922 (1.0%)
Chronic respiratory disease (not asthma)	4880 (3.3%)	3303 (8.4%)	1577 (1.5%)	2267 (4.5%)	2613 (2.7%)
Asthma with recent OCS use^d^	7558 (5.1%)	2597 (6.6%)	4961 (4.6%)	3120 (6.2%)	4438 (4.6%)
Asthma with no recent OCS use	14,861 (10.1%)	3582 (9.1%)	11,279 (10.5%)	5056 (10.1%)	9805 (10.1%)
Severe mental illness/antipsychotic use^a^	1700 (1.2%)	956 (2.4%)	744 (0.7%)	595 (1.2%)	1105 (1.1%)
Dementia	2407 (1.6%)	2046 (5.2%)	361 (0.3%)	1060 (2.1%)	1347 (1.4%)
Chronic neurological disease	1932 (1.3%)	1034 (2.6%)	898 (0.8%)	746 (1.5%)	1186 (1.2%)
Learning/intellectual disability	1014 (0.7%)	361 (0.9%)	653 (0.6%)	292 (0.6%)	722 (0.7%)
Non-haematological cancer					
Diagnosed <1 year ago	3839 (2.6%)	2275 (5.8%)	1564 (1.5%)	1807 (3.6%)	2032 (2.1%)
Diagnosed 1–4.9 years ago	4554 (3.1%)	2085 (5.3%)	2469 (2.3%)	1878 (3.8%)	2676 (2.8%)
Diagnosed ≥5 years ago	8436 (5.7%)	2764 (7.0%)	5672 (5.3%)	2998 (6.0%)	5438 (5.6%)
Haematological malignancy					
Diagnosed <1 year ago	558 (0.4%)	373 (0.9%)	185 (0.2%)	246 (0.5%)	312 (0.3%)
Diagnosed 1–4.9 years ago	273 (0.2%)	162 (0.4%)	111 (0.1%)	124 (0.2%)	149 (0.2%)
Diagnosed ≥5 years ago	278 (0.2%)	114 (0.3%)	164 (0.2%)	113 (0.2%)	165 (0.2%)
Rheumatoid arthritis^a^	1276 (0.9%)	648 (1.6%)	628 (0.6%)	560 (1.1%)	716 (0.7%)
Systemic lupus erythematosus^a^	164 (0.1%)	57 (0.1%)	107 (0.1%)	46 (0.1%)	118 (0.1%)
HIV^a^	234 (0.2%)	56 (0.1%)	178 (0.2%)	100 (0.2%)	134 (0.1%)
Immunosuppression^e^	1404 (1.0%)	643 (1.6%)	761 (0.7%)	597 (1.2%)	807 (0.8%)
Erectile dysfunction^a^	7183 (10.9%)	5293 (21.2%)	1890 (4.6%)	3556 (14.0%)	3627 (8.9%)

a In QRISK3 algorithm, but non-imputed version included here (for smoking status, cholesterol:HDL ratio, systolic BP and BMI).

b Most recent measure before baseline. N with missing cholesterol:HDL measurement 55,392 (37.7%).

c Used on hypertension definition. N with missing systolic BP measurement 16,713 (11.4%).

d At least 1 prescription in the 12 months before baseline. Other than corticosteroids which was defined as at least 2 prescriptions prior to baseline with the most recent ≤28 days before baseline.

e Ever history of solid organ transplant or permanent cellular immune deficiency; history in the 24 months before baseline for aplastic anaemia, bone marrow or stem cell transplant; history in the 12 months before baseline for biologics or other immunosuppressant therapy (excluding corticosteroids), other or unspecified cellular immune deficiency.

### Risk of death after COVID-19

**Fig. 3:**
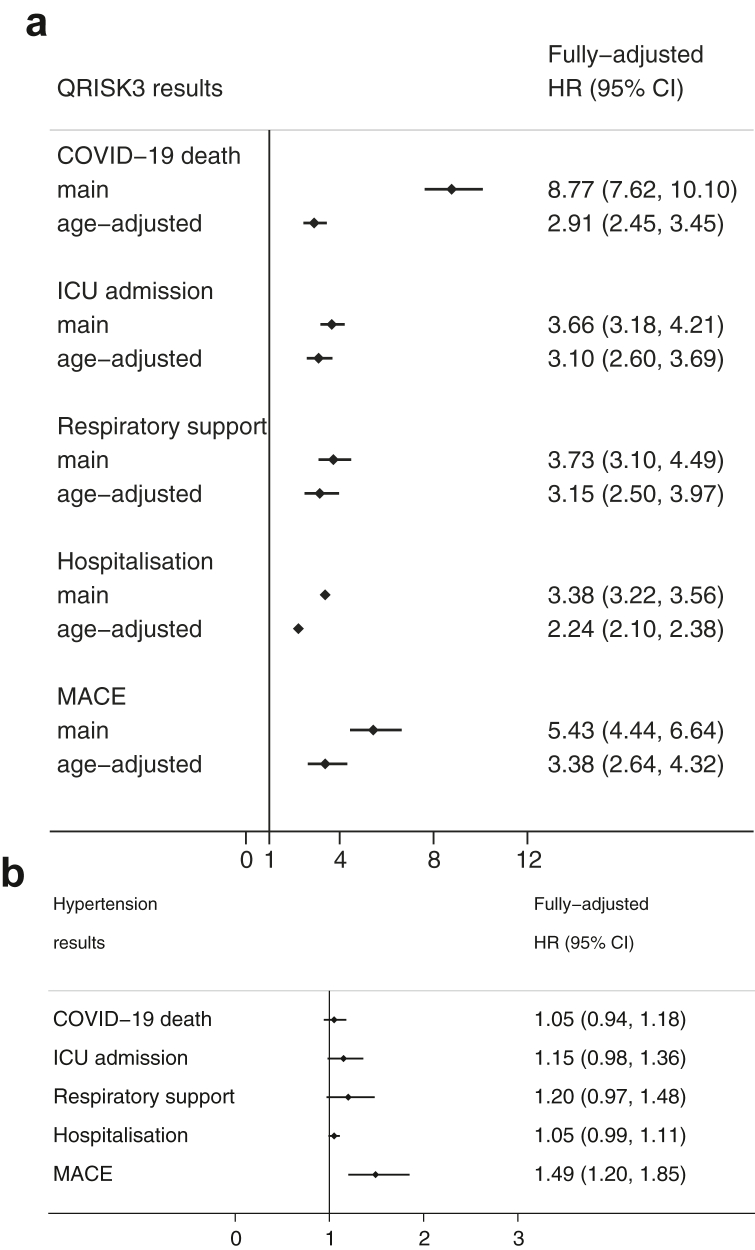
**Adjusted hazard ratios for raised cardiovascular risk effect on COVID-19 severe outcomes presented separately for a) QRISK3 and b) hypertension from complete case analysis.** QRISK3 score hazard ratios are for the effect of a score ≥ 10% with <10% as the reference (N = 97,725 for complete case analysis compared to 146,760 for crude model). Hypertension hazard ratios are for the effect of having hypertension with not having hypertension as the reference (N = 57,264 for complete case analysis). Hypertension models were adjusted for age, sex, ethnicity, socioeconomic status, body-mass index, alcohol consumption, smoking status, total cholesterol: high density lipoprotein cholesterol ratio, family history of coronary heart disease, treatment with corticosteroids, antiplatelets, or anticoagulants, diagnosis of atrial fibrillation, migraine, diabetes, chronic kidney disease stage 3–5, chronic liver disease, chronic lung disease, asthma, severe mental illness, dementia, chronic neurological disease, learning disability, or malignancy, and treatment or diagnosis of a immunosuppressive condition; and QRISK3 models were adjusted for alcohol consumption, treatment with antiplatelets or anticoagulants, diagnosis of chronic liver disease, chronic lung disease, asthma, dementia, chronic neurological disease, learning disability, or malignancy, and treatment or diagnosis of an immunosuppressive condition (which are not included in the QRISK3 algorithm).

In unadjusted analysis, raised QRISK3 score was associated with a substantial increase in COVID-19 death overall (HR 16.33 [14.61–18.24] N = 146,760) and in the study population with complete data available (HR 14.95 [13.07–17.10], N = 97,725) (Supplementary Table S6). After adjustment for non-QRISK3 confounders, the association between QRISK3 score and COVID-19 death attenuated but remained substantial (aHR 8.77 [7.62–10.10]). Characteristics of those included and excluded from the fully-adjusted model are shown in Supplementary Table S7. Among all patients aged 40–54 years, 6.1% had a QRISK3 score ≥10% but among those who died from COVID-19, 25.4% had a QRISK3 score ≥10% (Supplementary Table S8). In age-stratified and further age-adjusted analysis, the association between QRISK3 score and COVID-19 death was diminished compared to the main effect estimate but remained statistically significant in all age-group strata (Supplementary Table S9) with an age-adjusted result of 2.91 (2.45–3.45) In comparison, there was no association between hypertension and COVID-19 death (aHR 1.05 [0.94–1.18], N = 57,264) (Fig. 3). Results for all adjustment factors are shown in Supplementary Fig. S1.

### Risk of other severe outcomes after COVID-19

Significant associations were also found for QRISK3 score ≥10% and the outcomes of ICU admission (aHR 3.66 [3.18–4.21]), respiratory support (aHR 3.73 [3.10–4.49]), hospitalisation (aHR 3.38 [3.22–3.56]), and MACE (aHR 5.43 [4.44–6.64]). There was only a minor reduction in associations after further adjustment for age (Fig. 3). There was no association between hypertension and ICU admission (aHR 1.15 [0.98–1.36]), respiratory support (aHR 1.20 [0.97–1.48]), or hospitalisation (aHR 1.05 [0.99–1.11]) but there was an association between hypertension and MACE (aHR 1.49 [1.20–1.85]).

### Additional analyses

Results between wave 1 and wave 2 were broadly similar for all outcomes (Supplementary Table S10). Similar results were also obtained when COVID-19 was clinically reported rather than laboratory-confirmed (Supplementary Table S11). Further stratification of the QRISK3 score showed a substantially greater risk of COVID-19 death in individuals with a QRISK3 score of ≥20% (aHR 15.15 [13.05–17.59]) than 10-<20% (aHR 5.32 [4.54–6.23]) when both were compared to those with a score <10% (Supplementary Table S12). A similar, though less extreme gradient was observed for the other outcomes.

## Discussion

In this large, population-based cohort study using linked data from England in 2020, we found a striking gradient in the occurrence of severe COVID-19 outcomes by underlying cardiovascular risk profile among people without pre-existing CVD. The risks of death, ICU admission, hospital admission and MACE were all greater among individuals at raised cardiovascular risk measured by QRISK3 score, compared to those at low risk, despite no increase in recorded infections in this group. Associations between raised cardiovascular risk and COVID-19 deaths remained present, though attenuated, when results were stratified by 15-year age-group and further adjusted for age. When cardiovascular risk was measured by hypertension alone, differences were only evident for MACE outcomes. Analysis by pandemic waves revealed similar patterns, although the incidence of severe outcomes was greatest during the first wave.

Our study used linked electronic health record data from primary and secondary, including intensive, care, mortality records and national laboratory surveillance to capture detailed clinical and laboratory data on SARS-CoV-2 infections and outcomes. It is, to our knowledge, the first UK population-based study to assess COVID-19 outcomes using a comprehensive, combined measure of cardiovascular risk, QRISK3, rather than focusing on individual vascular risk factors. Findings from this large, representative cohort should be generalizable to adults in England aged 40–84 years (the upper age for which QRISK3 can be used to assess cardiovascular risk). Our dataset spanned the first and major part of the second wave of the COVID-19 pandemic in England, allowing comparisons of outcomes between waves. Limiting follow up to the end of December 2020 prevented contamination from the emergence of coronavirus variants or widespread roll out of the COVID-19 vaccination programme in England.

Nevertheless, differences in the availability of laboratory PCR testing are likely to have led to differences in the reported incidence of infection between waves: a laboratory-confirmed definition of SARS-CoV-2 lacked sensitivity to identify cases occurring during wave one before mass testing became widely available. It is also possible that some outcomes such as hospitalisation or MACE may have led to in-hospital testing, strengthening the observed association between vascular risk status and severe outcomes in the laboratory-confirmed cohort during the first wave. However, individuals who were at raised cardiovascular risk defined by QRISK3 score had a lower incidence of laboratory-confirmed infection than those at low cardiovascular risk, suggesting that differential in-hospital testing is unlikely to have biased our results. Reasons for the lower rates of laboratory-confirmed infections among individuals at high cardiovascular risk are unclear but may reflect reduced access to community testing e.g. due to shielding. Stratifying by pandemic wave to explore the effect of expanded testing and advances in clinical management of COVID-19 in later time periods revealed similar results to the main analysis. When we compared results for individuals with confirmed SARS-CoV-2 infection to those with clinically diagnosed COVID-19, we also saw similar patterns. Our descriptive analysis of COVID-19 outcomes alone regardless of recorded infection status supported findings from the cohort analysis.

The magnitude of association between cardiovascular risk status and severe outcomes varied by the method used to classify cardiovascular risk. In general, classification by QRISK3 produced more exaggerated differences between high and low cardiovascular risk groups than classification by hypertension alone. This is perhaps unsurprising as QRISK3 is a more comprehensive measure of cardiovascular risk, which includes additional comorbidities and socio-demographic components of risk. While age is a major driver of severe COVID-19 outcomes, associations with raised cardiovascular risk remained present in both age-stratified and age-adjusted analyses. Although misclassification of cardiovascular risk status could have occurred due to the documented reductions in GP visits and healthcare-seeking for non-COVID conditions during the pandemic,^23^ under-recognition of individuals at raised cardiovascular risk would have led to bias towards the null. In addition, our sensitivity analysis in which QRISK3 status was graded more finely into three strata (<10%, 10–19%, 20%+), confirmed a gradient of increasing risk of severe outcomes with increasing vascular risk level, which suggests that the main results are robust to any minor exposure misclassification. As QRISK3 scores were developed for the UK population, levels of cardiovascular risk identified in our study population may differ to those in other countries using different risk scores, although results of our hypertension analysis should generalize to other settings.

Residual confounding may also have been present in our study. While we adjusted for a broad range of sociodemographic, lifestyle and clinical confounding factors, some variables are either not measured (such as genetic risk profiles) or are sub-optimally recorded (such as BMI) in EHRs. Nevertheless, population-based self-controlled case series analyses of COVID-19 and thrombotic outcomes, which use within-person comparisons to control implicitly for fixed confounding^24^ show comparable results to cohort studies,^11^^,^^25^ suggesting that confounding is unlikely to explain our cohort results. Missing data on alcohol consumption reduced numbers for the QRISK3 complete case analysis (as other lifestyle factors were imputed in the QRISK3 algorithm if missing) whereas for hypertension, reduced numbers were driven by missing data on alcohol, smoking, BMI, cholesterol and ethnicity. Nevertheless, complete case analysis gives unbiased results when the chance of being a complete case is independent of outcome after taking covariates into account, even when data are missing not at random.^26^

Our findings extend those from previous smaller studies of individual cardiovascular risk factors and COVID-19 outcomes,^27^^,^^28^ supporting a strong association between raised cardiovascular risk profile and severe COVID-19 outcomes. While a previous Mendelian randomisation study, which by design avoids reverse causation and most confounding, failed to show an association between some genetically-predicted cardiovascular risk factors (blood pressure, BMI, type 2 diabetes and coronary artery disease) and COVID-19 hospitalisation,^29^ estimates had wide confidence intervals and did not capture full profiles of either cardiovascular risk or severe COVID-19 outcomes. The bidirectional relationship between cardiovascular risk and COVID-19 shown in our study is consistent with pre-COVID era work on cardiovascular complications of acute respiratory infections, showing a gradient in the risk of complications aligned with underlying cardiovascular risk status.^14^

Potential mechanisms underlying severe outcomes in COVID-19 include pro-inflammatory, pro-thrombotic and vasoconstrictive effects of SARS-CoV-2-mediated imbalances in ACE-2/RAS signalling.^30^ It has been suggested that individuals with conditions leading to raised cardiovascular risk are likely to have altered cytokine profiles leading to chronic systemic inflammation, which may have a synergistic effect on disease severity in acute COVID-19.^31^ A substantial burden of cardiovascular disease has also been demonstrated in survivors of acute COVID-19 at one year.^32^^,^^33^ Understanding the natural history of longer-term cardiovascular and other complications including post-COVID-19 syndrome^34^ in individuals at raised cardiovascular risk, along with the mechanisms underlying both short and long-term health changes, should be a priority for future research. Future studies could also investigate the role of COVID-19 treatments in modifying or mediating the relationship between raised cardiovascular risk and severe COVID-19. Combining mechanistic research with clinical evidence to improve patient care among those at raised cardiovascular risk is essential to prevent and manage severe outcomes of COVID-19 in this group.^35^ Our study highlights the need for a continued focus on integrated prevention e.g. combining COVID-19 vaccinations with cardiovascular disease prevention to improve health among those at raised cardiovascular risk.

In conclusion, we showed that individuals at raised cardiovascular risk in England were more likely to die or to experience severe outcomes after COVID-19 than those at low cardiovascular risk, despite not initially being identified as a vulnerable group. Those at raised cardiovascular risk should be considered a priority for targeted prevention and treatment strategies for COVID-19. Addressing cardiovascular risk factors could improve outcomes after COVID-19.

## Contributors

CWG conceptualized the study and obtained funding. CWG, JAD, HS, EH, LS, JB and AB contributed to study design. JAD managed and analysed data, supported by EH, HS and CWG. JAD and CWG drafted the manuscript. All authors reviewed the manuscript, interpreted data and approved the final version for publication.

## Data sharing statement

Data used for the study were obtained from the UK CPRD database under licence from the UK Medicines and Healthcare Products Regulatory Agency. Access to CPRD data is subject to protocol approval via CPRD's Research Data Governance Process (https://cprd.com/data-access). All codelists used for this study are available on LSHTM Data Compass: https://doi.org/10.17037/DATA.00002762. Analytical code is available via GitHub: https://github.com/jenAdavidson/cvrisk_covid_cohort.

## Ethical approval

The CPRD Independent Scientific Advisory Committee (application 20_000135) and the London School of Hygiene and Tropical Medicine (LSHTM) Ethics Committee (application 22717) approved the study.

## Declaration of interests

CWG reports participation in the data safety and monitoring board for the IAMI trial of influenza vaccine for cardiovascular disease (NCT02831608) ending April 2020. JB reports consulting fees from ARCbio, HVivo and GSK and participation in a data safety and monitoring board for the COM Cov trial, Oxford, now ended. AB reports grants from NIHR, AstraZeneca and the British Medical Association and leadership roles as Vice-President, Digital, Marketing, Communications for the British Cardiovascular Society and Senior Advisor to the Emerging Leaders Programme of the World Heart Federation. All other authors report no conflicts.
