# Supplementary material for: Severe COVID-19 outcomes by cardiovascular risk profile in England in 2020: a population-based cohort study
Source: Lancet Reg Health Eur. 2023 Mar 7;27:100604. doi: 10.1016/j.lanepe.2023.100604 (PMC9991014; doi:10.1016/j.lanepe.2023.100604)

Supplementary material

Contents

Supplementary methods 1. Defining cardiovascular risk using QRISK3 2

Supplementary methods 2. Covariates included in analysis 2

Supplementary references 3

Supplementary table 1. Baseline characteristics of the incidence study population by cardiovascular risk 4

Supplementary table 2. Number and incidence rate of laboratory-confirmed SARS-CoV-2 and clinically reported COVID-19 and outcomes of interest during wave one of the COVID-19 pandemic 7

Supplementary table 3. Number and incidence rate of laboratory-confirmed SARS-CoV-2 and clinically reported COVID-19 and outcomes of interest during wave two of the COVID-19 pandemic 8

Supplementary table 4. Age standardised incidence rates per 1,000 (with 95% confidence intervals) of laboratory-confirmed SARS-CoV-2 and clinically reported COVID-19 and outcomes of interest 9

Supplementary table 5. Baseline characteristics of the clinically reported COVID-19 study population by cardiovascular risk 11

Supplementary table 6. Hazard ratios for the effect of raised cardiovascular risk on severe outcomes after laboratory-confirmed SARS-CoV-2 from complete case analysis 14

Supplementary table 7. Baseline characteristics of those included in and excluded from complete case analysis for the laboratory-confirmed SARS-CoV-2 study population 16

Supplementary table 8. The number and proportion of patients with outcomes of interest by age group and QRISK3 score level 17

Supplementary table 9. Hazard ratios for the effect of raised QRISK3 score on severe outcomes after laboratory-confirmed SARS-CoV-2 with age-adjustment and age stratification from complete case analysis 18

Supplementary table 10. Hazard ratios for the effect of raised cardiovascular risk on severe outcomes after laboratory-confirmed SARS-CoV-2 from complete case analysis stratified by wave of the pandemic 19

Supplementary table 11. Hazard ratios for the effect of raised cardiovascular risk on severe outcomes after clinically reported COVID-19 from complete case analysis 21

Supplementary table 12. Hazard ratios for the effect of raised cardiovascular risk with refined QRISK3 score categories on severe outcomes after laboratory-confirmed SARS-CoV-2 from complete case analysis 22

Supplementary figure 1. Adjusted hazard ratios in hypertension model for the association between laboratory-confirmed SARS-CoV-2 and COVID-19 death from complete case analysis 23

Supplementary methods 1. Defining cardiovascular risk using QRISK3

QRISK3 is a validated UK ten-year cardiovascular risk prediction score for myocardial infarction or stroke based on a combination of known risk factors[1]. The risk score is calculated based on: age, sex, ethnicity, socio-economic status, family history of coronary heart disease in a first degree relative aged <60 years, body mass index (BMI), systolic blood pressure reading and its variability, total cholesterol to high density lipoprotein cholesterol ratio, smoking status, corticosteroid treatment, and the presence of comorbid health conditions. The comorbid health conditions included are: diabetes, treated hypertension, rheumatoid arthritis, systemic lupus erythematosus (SLE), atrial fibrillation, chronic kidney disease stages 3-5, migraine, severe mental illness, HIV, and in men, erectile dysfunction. We calculated individual score's using our Stata program[2] with selected codes and measures recorded in patient records prior to baseline for each of the conditions included in the calculator and with the weighted algorithm made available by the QRISK3 developers[3]. We considered the absence of a recorded comorbid condition to mean the patient did not have the condition and imputed missing lifestyle and anthropometric measures (such as BMI) with the average value for the study population, in line with how the algorithm used by general practitioners works in clinical practice.

Supplementary methods 2. Covariates included in analysis

Covariates differed depending on the whether QRISK3 or hypertension were used to define cardiovascular risk.

In analyses where cardiovascular risk was defined by QRISK3, we included covariates which were not part of determining the QRISK3 score. These variables were: alcohol consumption, treatment with antiplatelets or anticoagulants, diagnosis of chronic liver disease, chronic lung disease, asthma, dementia, chronic neurological disease, learning or intellectual disability, or malignancy, and treatment or diagnosis of a immunosuppressive condition (solid organ transplant or permanent cellular immunodeficiency, or aplastic anaemia, bone marrow or stem cell transplant, biologic or other immunosuppressant therapy, or temporary immunodeficiency). Additionally, we included age in further adjustment due to the strong association between age, COVID-19 and the risk of severe outcomes.

In analyses where hypertension was used to define cardiovascular risk, we included covariates accounted for in the QRISK3 algorithm as well as those no included in the algorithm but adjusted for in QRISK3 analysis. These variables were: baseline age, sex (male and female), ethnicity (White, south Asian, Black, and mixed or other), socioeconomic status (individual-level Townsend score grouped into quintiles, ranging from least deprived [quintile 1] to most deprived [quintile 5]), BMI (underweight [<18.5 kg/m2], normal [18.5-24.9 kg/m2], overweight [25.0-29.9 kg/m2], obese [30.0-39.9 kg/m2], and severely obese [≥40 kg/m2]), total cholesterol to high-density lipoprotein ratio, alcohol consumption (heavy drinking [defined as either a recorded intake of >42 units per week or a diagnostic code suggestive of alcohol addiction or excessive alcohol consumption] or no known heavy drinking), smoking status (current smoker, never smoker, or former smoker), family history of heart disease, and comorbid conditions or treatments which are potential risk factors for severe COVID-19 outcomes. These comorbidities and treatments were those included in the QRISK3 algorithm (diabetes, atrial fibrillation, migraine, chronic kidney disease stage 3-5, corticosteroid use, severe mental illness or antipsychotic use, and erectile dysfunction) as well as chronic respiratory diseases, asthma, non-haematological and haematological cancer, chronic liver disease, dementia, chronic neurological disease, learning or intellectual disability. Immunosuppression was also included as a

covariate, in which we grouped elements included in QRISK3 (rheumatoid arthritis, SLE and HIV) with other elements of any prior solid organ transplant or permanent cellular immunodeficiency, or aplastic anaemia, bone marrow or stem cell transplant recorded within the 24 months before index, or biologic or other immunosuppressant therapy (excluding corticosteroids) or temporary immunodeficiency recorded within the 12 months before index date.

Comorbid conditions were defined using coded CPRD Aurum data for any previous clinical diagnosis, major intervention for, or clinical review specific to the condition of interest. In addition, chronic liver disease and cancer were additionally defined using HES as recording in CPRD alone may underestimate the prevalence of the conditions[4, 5].

Supplementary references

1. Hippisley-Cox J, Coupland C, Brindle P. Development and validation of QRISK3 risk prediction algorithms to estimate future risk of cardiovascular disease: prospective cohort study. Bmj. 2017 May 23;357:j2099
2. Davidson JA, Strongman H, Herrett E, Gadd S. qrisk_cprd_aurum: QRISK Aurum bundle version 2.0 2022. Available from: https://zenodo.org/record/5822521#.YrrC6XbMKUl Last accessed 28/06/2022
3. ClinRisk Ltd. QRISK3 algorithm. 2017. Available from: https://qrisk.org/three/src.php Last accessed 28/06/2022
4. Strongman H, Williams R, Bhaskaran K. What are the implications of using individual and combined sources of routinely collected data to identify and characterise incident site-specific cancers? a concordance and validation study using linked English electronic health records data. BMJ Open. 2020 Aug 20;10(8):e037719
5. Walker JL, Grint DJ, Strongman H, Eggo RM, Peppa M, Minassian C, et al. UK prevalence of underlying conditions which increase the risk of severe COVID-19 disease: a point prevalence study using electronic health records. BMC Public Health. 2021 Mar 11;21(1):484

Supplementary table 1. Baseline characteristics of the incidence study population by cardiovascular risk

	All	Established CVD	QRISK3 score		Hypertension	
			Raised risk	Low risk	Raised risk	Low risk
	N=6,059,055	N=741,913	N=1,929,627	N=3,387,515	N=1,881,654	N=3,435,488
Age (years), Mean (SD)*	57.6 (12.1)	68.1 (10.7)	67.4 (8.9)	49.7 (7.1)	60.8 (11.6)	53.6 (10.7)
Age group (years)*						
40-54	2,759,591 (45.5%)	95,579 (12.9%)	167,366 (8.7%)	2,496,646 (73.7%)	623,005 (33.1%)	2,041,007 (59.4%)
55-64	1,488,257 (24.6%)	160,677 (21.7%)	526,344 (27.3%)	801,236 (23.7%)	526,946 (28.0%)	800,634 (23.3%)
65-74	1,125,307 (18.6%)	238,877 (32.2%)	796,797 (41.3%)	89,633 (2.6%)	459,164 (24.4%)	427,266 (12.4%)
75-84	685,900 (11.3%)	246,780 (33.3%)	439,120 (22.8%)	0 (0.0%)	272,539 (14.5%)	166,581 (4.8%)
Sex*						
Women	3,016,430 (49.8%)	303,691 (40.9%)	803,754 (41.7%)	1,908,985 (56.4%)	907,170 (48.2%)	1,805,569 (52.6%)
Men	3,042,578 (50.2%)	438,216 (59.1%)	1,125,832 (58.3%)	1,478,530 (43.6%)	974,469 (51.8%)	1,629,893 (47.4%)
Unknown	47 (0.0%)	6 (0.0%)	41 (0.0%)	0 (0.0%)	15 (0.0%)	26 (0.0%)
Ethnicity*						
White or not stated	4,520,954 (74.6%)	592,469 (79.9%)	1,525,861 (79.1%)	2,402,624 (70.9%)	1,441,447 (76.6%)	2,487,038 (72.4%)
South Asian	284,496 (4.7%)	34,849 (4.7%)	98,843 (5.1%)	150,804 (4.5%)	75,778 (4.0%)	173,869 (5.1%)
Black	122,348 (2.0%)	7,544 (1.0%)	16,844 (0.9%)	97,960 (2.9%)	42,751 (2.3%)	72,053 (2.1%)
Mixed/Other	408,571 (6.7%)	42,050 (5.7%)	95,746 (5.0%)	270,775 (8.0%)	123,701 (6.6%)	242,820 (7.1%)
Unknown	722,686 (11.9%)	65,001 (8.8%)	192,333 (10.0%)	465,352 (13.7%)	197,977 (10.5%)	459,708 (13.4%)
Townsend quintile*						
1 (least deprived)	1,377,227 (22.7%)	158,440 (21.4%)	442,185 (22.9%)	776,602 (22.9%)	440,270 (23.4%)	778,517 (22.7%)
2	1,270,784 (21.0%)	156,147 (21.0%)	414,389 (21.5%)	700,248 (20.7%)	407,867 (21.7%)	706,770 (20.6%)
3	1,153,335 (19.0%)	147,442 (19.9%)	368,505 (19.1%)	637,388 (18.8%)	361,033 (19.2%)	644,860 (18.8%)
4	1,075,138 (17.7%)	139,288 (18.8%)	336,485 (17.4%)	599,365 (17.7%)	327,735 (17.4%)	608,115 (17.7%)
5 (most deprived)	1,177,997 (19.4%)	140,093 (18.9%)	366,651 (19.0%)	671,253 (19.8%)	343,397 (18.2%)	694,507 (20.2%)
Unknown	4,574 (0.1%)	503 (0.1%)	1,412 (0.1%)	2,659 (0.1%)	1,352 (0.1%)	2,719 (0.1%)
Region of residence						
North East	195,161 (3.2%)	29,489 (4.0%)	67,385 (3.5%)	98,287 (2.9%)	65,030 (3.5%)	100,642 (2.9%)
North West	1,127,967 (18.6%)	163,745 (22.1%)	366,543 (19.0%)	597,679 (17.6%)	364,348 (19.4%)	599,874 (17.5%)
Yorkshire and the Humber	199,901 (3.3%)	26,077 (3.5%)	68,746 (3.6%)	105,078 (3.1%)	64,774 (3.4%)	109,050 (3.2%)
East Midlands	112,527 (1.9%)	13,988 (1.9%)	36,263 (1.9%)	62,276 (1.8%)	36,110 (1.9%)	62,429 (1.8%)

West Midlands	991,220 (16.4%)	127,718 (17.2%)	341,713 (17.7%)	521,789 (15.4%)	338,837 (18.0%)	524,665 (15.3%)
East of England	266,088 (4.4%)	27,946 (3.8%)	80,234 (4.2%)	157,908 (4.7%)	79,741 (4.2%)	158,401 (4.6%)
South West	1,155,120 (19.1%)	109,644 (14.8%)	321,836 (16.7%)	723,640 (21.4%)	319,626 (17.0%)	725,850 (21.1%)
South Central	1,275,851 (21.1%)	140,308 (18.9%)	403,213 (20.9%)	732,330 (21.6%)	381,420 (20.3%)	754,123 (22.0%)
London	725,286 (12.0%)	101,616 (13.7%)	240,047 (12.4%)	383,623 (11.3%)	228,485 (12.1%)	395,185 (11.5%)
Unknown	9,934 (0.2%)	1,382 (0.2%)	3,647 (0.2%)	4,905 (0.1%)	3,283 (0.2%)	5,269 (0.2%)
BMI category*†						
Underweight (<18·5 kg/m^2)	54,108 (0.9%)	9,692 (1.3%)	18,173 (0.9%)	26,243 (0.8%)	10,520 (0.6%)	33,896 (1.0%)
Normal (18·5-24·9 kg/m^2)	1,204,515 (19.9%)	145,084 (19.6%)	371,520 (19.3%)	687,911 (20.3%)	283,091 (15.0%)	776,340 (22.6%)
Overweight (25·0-29·9 kg/m^2)	1,554,717 (25.7%)	233,765 (31.5%)	580,257 (30.1%)	740,695 (21.9%)	516,934 (27.5%)	804,018 (23.4%)
Obese (30·0-39·9 kg/m^2)	1,191,415 (19.7%)	214,291 (28.9%)	465,390 (24.1%)	511,734 (15.1%)	499,738 (26.6%)	477,386 (13.9%)
Severely obese (≥40.0 kg/m^2)	171,358 (2.8%)	32,283 (4.4%)	62,506 (3.2%)	76,569 (2.3%)	83,298 (4.4%)	55,777 (1.6%)
Unknown	1,882,942 (31.1%)	106,798 (14.4%)	431,781 (22.4%)	1,344,363 (39.7%)	488,073 (25.9%)	1,288,071 (37.5%)
Cholesterol:HDL, Mean (SD)*†	3.6 (1.2)	3.4 (1.1)	3.8 (1.2)	3.6 (1.1)	3.7 (1.2)	3.7 (1.2)
Systolic blood pressure, Mean (SD)*†‡	132.4 (6176.4)	131.0 (186.4)	143.1 (10446.7)	125.5 (13.8)	140.1 (140.3)	127.8 (8547.6)
Smoking status*†						
Non-smoker	2,604,078 (43.0%)	303,876 (41.0%)	814,910 (42.2%)	1,485,292 (43.8%)	872,183 (46.4%)	1,428,019 (41.6%)
Ex-smoker	1,400,144 (23.1%)	280,913 (37.9%)	538,682 (27.9%)	580,549 (17.1%)	472,013 (25.1%)	647,218 (18.8%)
Current smoker	765,115 (12.6%)	105,704 (14.2%)	301,860 (15.6%)	357,551 (10.6%)	228,312 (12.1%)	431,099 (12.5%)
Unknown	1,289,718 (21.3%)	51,420 (6.9%)	274,175 (14.2%)	964,123 (28.5%)	309,146 (16.4%)	929,152 (27.0%)
Alcohol consumption†						
No heavy drinking	3,420,295 (56.4%)	541,921 (73.0%)	1,228,590 (63.7%)	1,649,784 (48.7%)	1,168,514 (62.1%)	1,709,860 (49.8%)
Heavy drinking	514,459 (8.5%)	62,349 (8.4%)	176,737 (9.2%)	275,373 (8.1%)	172,181 (9.2%)	279,929 (8.1%)
Unknown	2,124,301 (35.1%)	137,643 (18.6%)	524,300 (27.2%)	1,462,358 (43.2%)	540,959 (28.7%)	1,445,699 (42.1%)
Family history of CHD*	447,753 (7.4%)	43,028 (5.8%)	180,543 (9.4%)	224,182 (6.6%)	138,688 (7.4%)	266,037 (7.7%)
Consultation frequency in prior 12 months, Median (IQR)	4 (1-8)	8 (4-14)	5 (2-9)	2 (0-6)	4 (2-9)	3 (0-6)
Medication use$						
Regular corticosteroids*	75,385 (1.2%)	25,110 (3.4%)	38,463 (2.0%)	11,812 (0.3%)	24,988 (1.3%)	25,287 (0.7%)
Antihypertensives*	1,823,538 (30.1%)	528,503 (71.2%)	759,312 (39.4%)	535,723 (15.8%)	829,575 (44.1%)	465,460 (13.5%)
Statins	1,270,675 (21.0%)	451,780 (60.9%)	660,072 (34.2%)	158,823 (4.7%)	492,588 (26.2%)	326,307 (9.5%)
Antiplatelets	698,994 (11.5%)	409,772 (55.2%)	211,453 (11.0%)	77,769 (2.3%)	163,743 (8.7%)	125,479 (3.7%)
Anticoagulants	237,101 (3.9%)	122,558 (16.5%)	86,070 (4.5%)	28,473 (0.8%)	59,399 (3.2%)	55,144 (1.6%)

Comorbid condition						
Atrial fibrillation*	162,317 (2.7%)	97,233 (13.1%)	61,788 (3.2%)	3,296 (0.1%)	36,815 (2.0%)	28,269 (0.8%)
Migraines*	159,222 (2.6%)	17,573 (2.4%)	33,288 (1.7%)	108,361 (3.2%)	42,268 (2.2%)	99,381 (2.9%)
Diabetes*	534,471 (8.8%)	163,196 (22.0%)	320,106 (16.6%)	51,169 (1.5%)	206,788 (11.0%)	164,487 (4.8%)
CKD stage 3-5*	619,694 (10.2%)	233,756 (31.5%)	325,064 (16.9%)	60,874 (1.8%)	241,312 (12.8%)	144,626 (4.2%)
Chronic liver disease	64,828 (1.1%)	18,131 (2.4%)	23,261 (1.2%)	23,436 (0.7%)	19,150 (1.0%)	27,547 (0.8%)
Chronic respiratory disease (not asthma)	302,464 (5.0%)	106,770 (14.4%)	147,122 (7.6%)	48,572 (1.4%)	94,365 (5.0%)	101,329 (2.9%)
Asthma with recent OCS use[$]	294,042 (4.9%)	57,789 (7.8%)	110,528 (5.7%)	125,725 (3.7%)	102,130 (5.4%)	134,123 (3.9%)
Asthma with no recent OCS use	539,665 (8.9%)	70,166 (9.5%)	143,006 (7.4%)	326,493 (9.6%)	160,391 (8.5%)	309,108 (9.0%)
Severe mental illness / antipsychotic use*	71,742 (1.2%)	12,428 (1.7%)	30,410 (1.6%)	28,904 (0.9%)	19,970 (1.1%)	39,344 (1.1%)
Dementia	86,965 (1.4%)	34,969 (4.7%)	42,323 (2.2%)	9,673 (0.3%)	22,768 (1.2%)	29,228 (0.9%)
Chronic neurological disease	97,037 (1.6%)	26,735 (3.6%)	37,752 (2.0%)	32,550 (1.0%)	28,232 (1.5%)	42,070 (1.2%)
Learning / intellectual disability	27,561 (0.5%)	3,620 (0.5%)	7,933 (0.4%)	16,008 (0.5%)	7,176 (0.4%)	16,765 (0.5%)
Non-haematological cancer						
Diagnosed <1 year ago	180,797 (3.0%)	47,481 (6.4%)	87,792 (4.5%)	45,524 (1.3%)	64,248 (3.4%)	69,068 (2.0%)
Diagnosed 1-4.9 years ago	238,061 (3.9%)	54,265 (7.3%)	112,826 (5.8%)	70,970 (2.1%)	83,171 (4.4%)	100,625 (2.9%)
Diagnosed ≥5 years ago	390,468 (6.4%)	68,501 (9.2%)	158,495 (8.2%)	163,472 (4.8%)	130,151 (6.9%)	191,816 (5.6%)
Haematological malignancy						
Diagnosed <1 year ago	20,007 (0.3%)	6,322 (0.9%)	9,781 (0.5%)	3,904 (0.1%)	6,475 (0.3%)	7,210 (0.2%)
Diagnosed 1-4.9 years ago	16,610 (0.3%)	4,622 (0.6%)	8,029 (0.4%)	3,959 (0.1%)	5,427 (0.3%)	6,561 (0.2%)
Diagnosed ≥5 years ago	14,591 (0.2%)	3,187 (0.4%)	6,073 (0.3%)	5,331 (0.2%)	4,577 (0.2%)	6,827 (0.2%)
Rheumatoid arthritis*	61,299 (1.0%)	13,551 (1.8%)	29,068 (1.5%)	18,680 (0.6%)	21,866 (1.2%)	25,882 (0.8%)
Systemic lupus erythematosus*	6,897 (0.1%)	1,581 (0.2%)	2,487 (0.1%)	2,829 (0.1%)	2,006 (0.1%)	3,310 (0.1%)
HIV*	7,910 (0.1%)	703 (0.1%)	1,913 (0.1%)	5,294 (0.2%)	2,460 (0.1%)	4,747 (0.1%)
Immunosuppression#	70,397 (1.2%)	16,191 (2.2%)	29,410 (1.5%)	24,796 (0.7%)	24,119 (1.3%)	30,087 (0.9%)
Erectile dysfunction*	323,765 (10.6%)	84,360 (19.3%)	186,601 (16.6%)	52,804 (3.6%)	120,368 (12.4%)	119,037 (7.3%)

* in QRISK3 algorithm, but non-imputed version included here (for smoking status, cholesterol:HDL ratio, systolic BP and BMI)
† most recent measure before baseline. N with missing cholesterol:HDL measurement 2,373,726 (39.2%)
‡ used on hypertension definition. N with missing systolic BP measurement 900,956 (14.9%)
$ at least 1 prescription in the 12 months before baseline. Other than corticosteroids which was defined as at least 2 prescriptions prior to baseline with the most recent ≤28 days before baseline
# ever history of solid organ transplant or permanent cellular immune deficiency; history in the 24 months before baseline for aplastic anaemia, bone marrow or stem cell transplant; history in the 12 months before baseline for biologics or other immunosuppressant therapy (excluding corticosteroids), other or unspecified cellular immune deficiency

Supplementary table 2. Number and incidence rate of laboratory-confirmed SARS-CoV-2 and clinically reported COVID-19 and outcomes of interest during wave one of the COVID-19 pandemic

	All		Established CVD		QRISK3 score				Hypertension			
					Raised risk		Low risk		Raised risk		Low risk	
	N	Rate (95% CI) per 1,000	N	Rate (95% CI) per 1,000	N	Rate (95% C.I.) per 1,000	N	Rate (95% CI) per 1,000	N	Rate (95% C.I.) per 1,000	N	Rate (95% CI) per 1,000
All individuals	5,955,940		731,214		1,903,772		3,320,954		1,854,236		3,370,490	
COVID-19 death*	4,653	1.9 (1.9-2.0)	2,440	8.1 (7.7-8.4)	1,908	2.4 (2.3-2.5)	305	0.2 (0.2-0.3)	1,155	1.5 (1.4-1.6)	1,058	0.8 (0.7-0.8)
Hospitalization$	13,757	5.6 (5.5-5.7)	5,579	18.2 (17.7-18.7)	5,217	6.5 (6.3-6.7)	2,961	2.2 (2.1-2.3)	4,013	5.1 (5.0-5.3)	4,165	3.0 (2.9-3.1)
Major adverse cardiovascular event	39,725	16.3 (16.1-16.4)	28,882	95.4 (94.3-96.5)	8,310	10.4 (10.2-10.7)	2,533	1.9 (1.8-2.0)	6,303	8.2 (8.0-8.4)	4,540	3.3 (3.2-3.4)
Laboratory-confirmed SARS-CoV-2	26,708	11.0 (10.8-11.1)	7,059	23.3 (22.8-23.9)	7,574	9.5 (9.3-9.7)	12,075	9.0 (8.9-9.2)	7,750	10.1 (9.8-10.3)	11,899	8.7 (8.6-8.9)
COVID-19 death*	3,525	50.3 (48.7-52.0)	1,881	196.3 (187.6-205.4)	1,424	84.5 (80.2-89.0)	220	5.0 (4.4-5.8)	885	42.5 (39.8-45.4)	759	19.1 (17.8-20.5)
ICU admission†	1,277	18.2 (17.3-19.3)	300	31.3 (28.0-35.1)	591	35.1 (32.3-38.0)	386	8.8 (8.0-9.8)	527	25.3 (23.3-27.6)	450	11.3 (10.3-12.4)
Respiratory support‡	815	11.6 (10.9-12.5)	161	16.8 (14.4-19.6)	395	23.4 (21.2-25.9)	259	5.9 (5.3-6.7)	365	17.5 (15.8-19.4)	289	7.3 (6.5-8.2)
Hospitalization$	7,794	111.2 (108.8-113.7)	3,071	320.5 (309.4-332.0)	3,032	179.8 (173.5-186.3)	1,691	38.8 (37.0-40.6)	2,349	112.9 (108.4-117.6)	2,374	59.8 (57.5-62.3)
Major adverse cardiovascular event	1,026	14.6 (13.8-15.6)	658	68.7 (63.6-74.1)	277	16.4 (14.6-18.5)	91	2.1 (1.7-2.6)	213	10.2 (9.0-11.7)	155	3.9 (3.3-4.6)
Clinically reported COVID-19	41,151	16.8 (16.7-17.0)	8,597	28.4 (27.8-29.0)	12,242	15.4 (15.1-15.6)	20,312	15.2 (15.0-15.4)	12,608	16.4 (16.1-16.6)	19,946	14.6 (14.4-14.8)
COVID-19 death*	584	20.1 (18.5-21.8)	293	52.8 (47.1-59.2)	257	30.7 (27.2-34.7)	34	2.2 (1.6-3.1)	135	15.2 (12.8-18.0)	156	10.6 (9.1-12.5)
Hospitalization$	2,421	83.2 (80.0-86.6)	872	157.1 (147.0-167.9)	925	110.4 (103.5-117.8)	624	41.1 (38.0-44.5)	749	84.2 (78.4-90.5)	800	54.6 (51.0-58.5)
Major adverse cardiovascular event	1,161	39.9 (37.7-42.3)	757	136.4 (127.0-146.5)	316	37.7 (33.8-42.1)	88	5.8 (4.7-7.2)	244	27.4 (24.2-31.1)	160	10.9 (9.4-12.8)

* ascertained from ONS death certificate data in which the COVID related ICD-10 codes U07.1 or U07.2 were present in the record

$ ascertained from presence in CHESS dataset or HES APC record coded with primary diagnosis of U07.1 or U07.2

† ascertained from CHESS records coded with ICU/HDU admission, only available for those with laboratory confirmed SARS-CoV-2

‡ ascertained from CHESS record coded with use of respiratory support via invasive mechanical ventilation, only available for those with laboratory confirmed SARS-CoV-2

Supplementary table 3. Number and incidence rate of laboratory-confirmed SARS-CoV-2 and clinically reported COVID-19 and outcomes of interest during wave two of the COVID-19 pandemic

	**All**		**Established CVD**		**QRISK3 score**				**Hypertension**			
					**Raised risk**		**Low risk**		**Raised risk**		**Low risk**	
	N	Rate (95% CI) per 1,000	N	Rate (95% CI) per 1,000	N	Rate (95% C.I.) per 1,000	N	Rate (95% CI) per 1,000	N	Rate (95% C.I.) per 1,000	N	Rate (95% CI) per 1,000
All individuals	5,862,260		707,279		1,865,383		3,289,598		1,824,647		3,330,334	
COVID-19 death*	3,213	0.7 (0.7-0.7)	1,724	3.1 (2.9-3.2)	1,295	0.9 (0.8-0.9)	194	0.1 (0.1-0.1)	859	0.6 (0.6-0.6)	630	0.2 (0.2-0.3)
Hospitalization$	14,256	3.1 (3.0-3.2)	5,301	9.4 (9.2-9.7)	5,577	3.8 (3.7-3.9)	3,378	1.3 (1.3-1.4)	4,468	3.1 (3.0-3.2)	4,487	1.7 (1.7-1.8)
Major adverse cardiovascular event	31,310	6.8 (6.7-6.9)	20,436	36.3 (35.8-36.8)	8,294	5.6 (5.5-5.7)	2,580	1.0 (1.0-1.1)	6,330	4.4 (4.3-4.5)	4,544	1.8 (1.7-1.8)
Laboratory-confirmed SARS-CoV-2	147,421	32.0 (31.9-32.2)	17,720	31.5 (31.0-32.0)	33,842	22.8 (22.5-23.0)	95,859	37.6 (37.3-37.8)	43,104	29.8 (29.6-30.1)	86,597	33.4 (33.2-33.6)
COVID-19 death*	2,950	22.2 (21.4-23.1)	1,612	92.0 (87.7-96.6)	1,173	37.4 (35.3-39.6)	165	2.0 (1.7-2.3)	779	19.9 (18.5-21.3)	559	7.4 (6.8-8.0)
ICU admission†	747	5.6 (5.2-6.0)	199	11.4 (9.9-13.1)	339	10.8 (9.7-12.0)	209	2.5 (2.2-2.9)	284	7.2 (6.4-8.1)	264	3.5 (3.1-3.9)
Respiratory support‡	269	2.0 (1.8-2.3)	69	3.9 (3.1-5.0)	131	4.2 (3.5-5.0)	69	0.8 (0.7-1.0)	110	2.8 (2.3-3.4)	90	1.2 (1.0-1.5)
Hospitalization$	10,099	76.1 (74.6-77.6)	3,484	198.9 (192.4-205.7)	4,015	128.0 (124.1-132.0)	2,600	31.0 (29.9-32.2)	3,279	83.7 (80.8-86.6)	3,336	43.9 (42.5-45.4)
Major adverse cardiovascular event	1,225	9.2 (8.7-9.8)	764	43.6 (40.6-46.8)	339	10.8 (9.7-12.0)	122	1.5 (1.2-1.7)	273	7.0 (6.2-7.8)	188	2.5 (2.1-2.9)
Clinically reported COVID-19	29,549	6.4 (6.4-6.5)	5,071	9.0 (8.8-9.3)	7,998	5.4 (5.3-5.5)	16,480	6.5 (6.4-6.6)	8,766	6.1 (5.9-6.2)	15,712	6.1 (6.0-6.2)
COVID-19 death*	139	2.6 (2.2-3.0)	72	7.1 (5.7-9.0)	61	4.0 (3.1-5.1)	6	0.2 (0.1-0.5)	43	2.6 (1.9-3.5)	24	0.9 (0.6-1.3)
Hospitalization$	1,271	23.5 (22.3-24.8)	464	45.9 (41.9-50.3)	460	29.8 (27.2-32.7)	347	12.2 (10.9-13.5)	375	22.7 (20.5-25.2)	432	15.7 (14.3-17.3)
Major adverse cardiovascular event	841	15.6 (14.5-16.7)	525	52.0 (47.7-56.6)	253	16.4 (14.5-18.6)	63	2.2 (1.7-2.8)	174	10.5 (9.1-12.2)	142	5.2 (4.4-6.1)

* ascertained from ONS death certificate data in which the COVID related ICD-10 codes U07.1 or U07.2 were present in the record

$ ascertained from presence in CHESS dataset or HES APC record coded with primary diagnosis of U07.1 or U07.2

† ascertained from CHESS records coded with ICU/HDU admission, only available for those with laboratory confirmed SARS-CoV-2

‡ ascertained from CHESS record coded with use of respiratory support via invasive mechanical ventilation, only available for those with laboratory confirmed SARS-CoV-2

Supplementary table 4. Age standardised incidence rates per 1,000 (with 95% confidence intervals) of laboratory-confirmed SARS-CoV-2 and clinically reported COVID-19 and outcomes of interest

		All	Established CVD	QRISK3 score		Hypertension	
				Raised risk	Low risk	Raised risk	Low risk
COVID-19 death*	All	1.44 (1.37-1.52)	3.21 (3.10-3.32)	1.16 (1.09-1.23)	0.24 (0.21-0.27)	0.97 (0.91-1.03)	0.97 (0.91-1.03)
	Women	1.08 (1.02-1.15)	2.84 (2.74-2.95)	1.01 (0.94-1.07)	0.19 (0.16-0.21)	0.71 (0.66-0.77)	0.78 (0.72-0.83)
	Men	1.81 (1.73-1.90)	3.43 (3.32-3.55)	1.37 (1.30-1.45)	6.06 (5.91-6.21)	1.27 (1.20-1.34)	1.21 (1.14-1.28)
Hospitalization$	All	4.93 (4.79-5.07)	11.09 (10.88-11.30)	5.77 (5.62-5.92)	2.06 (1.97-2.15)	4.34 (4.21-4.47)	3.39 (3.28-3.50)
	Women	4.00 (3.87-4.12)	10.48 (10.28-10.68)	6.30 (6.14-6.46)	1.89 (1.80-1.97)	3.74 (3.62-3.86)	2.76 (2.65-2.86)
	Men	5.89 (5.74-6.04)	11.46 (11.25-11.67)	6.04 (5.89-6.20)	8.33 (8.15-8.51)	5.02 (4.88-5.16)	4.16 (4.04-4.29)
Major adverse cardiovascular event	All	12.77 (12.55-13.00)	57.03 (56.57-57.50)	7.82 (7.65-799)	1.76 (1.68-1.84)	6.25 (6.10-6.41)	4.03 (3.91-4.16)
	Women	9.46 (9.27-9.65)	47.47 (47.05-47.90)	6.95 (6.78-7.11)	1.49 (1.42-1.57)	4.88 (4.74-5.01)	3.17 (3.06-3.28)
	Men	16.15 (15.90-16.40)	63.17 (62.68-63.66)	8.45 (8.27-8.63)	2.11 (2.02-2.20)	7.59 (7.42-7.76)	5.09 (4.95-5.23)
Laboratory-confirmed SARS-CoV-2	All	28.21 (27.88-28.54)	35.66 (35.29-36.03)	29.32 (28.98-29.65)	26.82 (26.50-27.14)	28.40 (28.07-28.73)	25.81 (25.50-26.13)
	Women	29.90 (29.56-30.24)	37.06 (36.68-37.43)	33.79 (33.43-34.15)	29.69 (29.35-30.03)	30.71 (30.37-31.06)	27.83 (27.50-28.15)
	Men	26.62 (26.30-26.94)	34.75 (34.39-35.12)	28.72 (28.39-29.05)	30.85 (30.51-31.20)	26.92 (26.60-27.24)	23.66 (23.36-23.96)
COVID-19 death*	All	51.84 (51.40-52.29)	80.91 (80.35-81.47)	45.75 (45.33-46.17)	10.93 (10.73-11.14)	42.16 (41.75-42.56)	39.89 (39.50-40.28)
	Women	41.58 (41.18-41.98)	72.63 (72.10-73.16)	37.86 (37.48-38.25)	7.90 (7.73-8.08)	32.26 (31.90-32.61)	32.70 (32.34-33.05)
	Men	60.93 (60.45-61.42)	85.79 (85.22-86.37)	53.10 (52.65-53.55)	28.77 (28.44-29.10)	52.30 (51.85-52.75)	48.13 (47.70-48.56)
ICU admission†	All	14.17 (13.93-14.40)	21.82 (21.53-22.11)	20.62 (20.34-20.90)	11.53 (11.32-11.74)	17.18 (16.92-17.44)	11.32 (11.11-11.53)
	Women	8.28 (8.10-8.46)	14.34 (14.10-14.57)	14.25 (14.01-14.48)	10.98 (10.77-11.18)	10.17 (9.97-10.37)	6.74 (6.58-6.90)
	Men	19.98 (19.70-20.25)	26.65 (26.33-26.97)	25.18 (24.87-25.49)	16.48 (16.23-16.74)	24.14 (23.84-24.45)	16.85 (16.60-17.11)
Respiratory support‡	All	7.50 (7.33-7.67)	11.21 (11.00-11.42)	11.79 (11.58-12.01)	8.88 (8.68-9.06)	10.07 (9.88-10.27)	5.73 (5.58-5.88)
	Women	3.99 (3.89-4.11)	7.28 (7.12-7.45)	8.19 (8.01-8.37)	8.72 (8.54-8.91)	5.52 (5.37-5.66)	3.12 (3.01-3.23)
	Men	11.00 (10.80-11.21)	13.66 (13.43-13.89)	14.49 (14.26-14.73)	13.14 (12.91-13.36)	14.59 (14.35-14.82)	8.97 (8.78-9.15)
Hospitalization$	All	130.28 (129.57-130.99)	201.81 (200.03-202.69)	151.09 (150.33-151.85)	55.66 (55.19-56.12)	126.63 (125.93-127.33)	106.61 (105.97-107.25)
	Women	108.39 (107.74-109.03)	187.47 (186.62-188.32)	139.20 (138.47-139.93)	47.62 (47.19-48.05)	106.00 (105.37-106.64)	87.33 (86.75-87.91)
	Men	151.87 (151.11-152.64)	210.26 (209.36-211.15)	162.61 (161.82-163.40)	94.26 (93.66-94.87)	147.23 (146.48-147.98)	130.09 (129.38-130.80)

		All	Established CVD	QRISK3 score		Hypertension	
				Raised risk	Low risk	Raised risk	Low risk
Major adverse cardiovascular event	All	17.43 (17.17-17.69)	43.35 (43.04-43.86)	12.10 (11.88-12.31)	2.87 (2.76-2.97)	10.94 (10.73-11.14)	7.51 (7.34-7.68)
	Women	13.56 (13.33-13.79)	37.56 (37.18-37.94)	9.69 (9.50-9.88)	2.49 (2.39-2.58)	7.80 (7.63-7.98)	5.99 (5.84-6.14)
	Men	21.12 (20.84-21.41)	47.03 (46.61-47.46)	13.58 (13.36-13.81)	2.77 (2.67-2.87)	13.97 (13.74-14.20)	9.31 (9.12-9.50)
Clinically reported COVID-19	All	11.74 (11.53-11.95)	18.84 (18.57-19.11)	13.35 (13.13-13.58)	10.23 (10.03-10.42)	11.68 (11.47-11.89)	10.14 (9.95-10.34)
	Women	13.39 (13.17-13.62)	23.09 (22.79-23.39)	19.06 (18.79-19.33)	12.04 (11.82-12.25)	14.07 (13.84-14.30)	11.63 (11.41-11.84)
	Men	10.13 (9.93-10.32)	16.14 (15.89-16.39)	11.97 (11.76-12.19)	7.68 (7.51-7.85)	9.84 (9.64-10.04)	8.49 (8.31-8.67)
COVID-19 death*	All	10.83 (10.62-11.03)	14.88 (14.64-15.12)	10.24 (10.05-10.44)	1.25 (1.18-1.32)	8.45 (8.27-8.63)	11.34 (11.13-11.55)
	Women	7.46 (7.29-7.63)	10.23 (10.03-10.43)	7.31 (7.15-7.48)	0.98 (0.92-1.04)	5.36 (5.22-5.51)	9.24 (9.05-9.42)
	Men	14.57 (14.33-14.80)	18.72 (18.46-18.99)	13.18 (12.95-13.40)	1.60 (1.52-1.68)	12.64 (12.42-12.86)	14.01 (13.78-14.24)
Hospitalization$	All	54.74 (54.28-55.20)	79.99 (79.44-80.55)	61.47 (60.98-61.95)	34.30 (33.94-34.66)	52.61 (52.16-53.05)	47.21 (46.78-47.63)
	Women	41.10 (40.70-41.50)	66.62 (66.11-67.12)	47.40 (46.97-47.83)	29.79 (29.45-30.13)	41.13 (40.74-41.53)	36.55 (36.18-36.93)
	Men	71.37 (70.85-71.90)	91.16 (90.57-91.75)	71.83 (71.30-72.35)	48.59 (48.16-49.02)	67.42 (66.91-67.93)	63.52 (63.03-64.01)
Major adverse cardiovascular event	All	30.58 (30.24-30.92)	76.39 (75.84-76.93)	22.52 (22.22-22.81)	4.26 (4.13-4.39)	20.03 (19.76-20.31)	14.64 (14.40-14.87)
	Women	24.21 (23.91-24.52)	72.69 (72.16-73.22)	17.99 (17.73-18.26)	3.80 (3.68-3.92)	14.09 (13.85-14.32)	11.56 (11.34-11.77)
	Men	37.96 (37.58-38.34)	77.97 (77.42-78.52)	25.72 (25.41-26.04)	5.40 (5.25-5.54)	27.24 (26.91-27.56)	18.58 (18.31-18.84)

* ascertained from ONS death certificate data in which the COVID related ICD-10 codes U07.1 or U07.2 were present in the record
$ ascertained from presence in CHESS dataset or HES APC record coded with primary diagnosis of U07.1 or U07.2
† ascertained from CHESS records coded with ICU/HDU admission, only available for those with laboratory confirmed SARS-CoV-2
‡ ascertained from CHESS record coded with use of respiratory support via invasive mechanical ventilation, only available for those with laboratory confirmed SARS-CoV-2

Supplementary table 5. Baseline characteristics of the clinically reported COVID-19 study population by cardiovascular risk

	All	QRISK3 score		Hypertension	
		**Raised risk**	**Low risk**	**Raised risk**	**Low risk**
	N=56,197	N=19,528	N=36,669	N=21,417	N=34,780
Age (years), Mean (SD)*	56.0 (10.9)	66.4 (9.6)	50.5 (6.7)	59.7 (11.2)	53.8 (10.1)
Age group (years)*					
40-54	28,447 (50.6%)	2,303 (11.8%)	26,144 (71.3%)	7,933 (37.0%)	20,514 (59.0%)
55-64	15,755 (28.0%)	6,114 (31.3%)	9,641 (26.3%)	6,629 (31.0%)	9,126 (26.2%)
65-74	7,627 (13.6%)	6,743 (34.5%)	884 (2.4%)	4,133 (19.3%)	3,494 (10.0%)
75-84	4,368 (7.8%)	4,368 (22.4%)	0 (0.0%)	2,722 (12.7%)	1,646 (4.7%)
Sex*					
Women	33,566 (59.7%)	8,722 (44.7%)	24,844 (67.8%)	11,952 (55.8%)	21,614 (62.1%)
Men	22,628 (40.3%)	10,803 (55.3%)	11,825 (32.2%)	9,463 (44.2%)	13,165 (37.9%)
Unknown	3 (0.0%)	3 (0.0%)	0 (0.0%)	2 (0.0%)	1 (0.0%)
Ethnicity*					
White or not stated	39,786 (70.8%)	14,577 (74.6%)	25,209 (68.7%)	15,252 (71.2%)	24,534 (70.5%)
South Asian	4,781 (8.5%)	1,993 (10.2%)	2,788 (7.6%)	1,623 (7.6%)	3,158 (9.1%)
Black	1,790 (3.2%)	282 (1.4%)	1,508 (4.1%)	765 (3.6%)	1,025 (2.9%)
Mixed/Other	5,500 (9.8%)	1,432 (7.3%)	4,068 (11.1%)	2,210 (10.3%)	3,290 (9.5%)
Unknown	4,340 (7.7%)	1,244 (6.4%)	3,096 (8.4%)	1,567 (7.3%)	2,773 (8.0%)
Townsend quintile*					
1 (least deprived)	10,083 (17.9%)	3,224 (16.5%)	6,859 (18.7%)	3,807 (17.8%)	6,276 (18.0%)
2	9,865 (17.6%)	3,309 (16.9%)	6,556 (17.9%)	3,845 (18.0%)	6,020 (17.3%)
3	10,042 (17.9%)	3,420 (17.5%)	6,622 (18.1%)	3,848 (18.0%)	6,194 (17.8%)
4	10,881 (19.4%)	3,781 (19.4%)	7,100 (19.4%)	4,077 (19.0%)	6,804 (19.6%)
5 (most deprived)	15,290 (27.2%)	5,784 (29.6%)	9,506 (25.9%)	5,823 (27.2%)	9,467 (27.2%)
Unknown	36 (0.1%)	10 (0.1%)	26 (0.1%)	17 (0.1%)	19 (0.1%)
Region of residence					
North East	1,236 (2.2%)	474 (2.4%)	762 (2.1%)	516 (2.4%)	720 (2.1%)
North West	11,964 (21.3%)	4,340 (22.2%)	7,624 (20.8%)	4,845 (22.6%)	7,119 (20.5%)
Yorkshire and the Humber	1,679 (3.0%)	652 (3.3%)	1,027 (2.8%)	650 (3.0%)	1,029 (3.0%)
East Midlands	778 (1.4%)	255 (1.3%)	523 (1.4%)	305 (1.4%)	473 (1.4%)
West Midlands	7,879 (14.0%)	2,853 (14.6%)	5,026 (13.7%)	3,178 (14.8%)	4,701 (13.5%)

East of England	1,893 (3.4%)	584 (3.0%)	1,309 (3.6%)	689 (3.2%)	1,204 (3.5%)
South West	14,852 (26.4%)	4,874 (25.0%)	9,978 (27.2%)	5,245 (24.5%)	9,607 (27.6%)
South Central	9,054 (16.1%)	3,027 (15.5%)	6,027 (16.4%)	3,282 (15.3%)	5,772 (16.6%)
London	6,793 (12.1%)	2,448 (12.5%)	4,345 (11.8%)	2,686 (12.5%)	4,107 (11.8%)
Unknown	69 (0.1%)	21 (0.1%)	48 (0.1%)	21 (0.1%)	48 (0.1%)
BMI category*†					
Underweight (<18·5 kg/m^2)	598 (1.1%)	285 (1.5%)	313 (0.9%)	159 (0.7%)	439 (1.3%)
Normal (18·5-24·9 kg/m^2)	11,078 (19.7%)	3,776 (19.3%)	7,302 (19.9%)	3,014 (14.1%)	8,064 (23.2%)
Overweight (25·0-29·9 kg/m^2)	15,335 (27.3%)	5,919 (30.3%)	9,416 (25.7%)	5,921 (27.6%)	9,414 (27.1%)
Obese (30·0-39·9 kg/m^2)	13,824 (24.6%)	5,722 (29.3%)	8,102 (22.1%)	6,824 (31.9%)	7,000 (20.1%)
Severely obese (≥40.0 kg/m^2)	2,588 (4.6%)	1,018 (5.2%)	1,570 (4.3%)	1,508 (7.0%)	1,080 (3.1%)
Unknown	12,774 (22.7%)	2,808 (14.4%)	9,966 (27.2%)	3,991 (18.6%)	8,783 (25.3%)
Cholesterol:HDL, Mean (SD)*†	3.7 (1.2)	3.8 (1.3)	3.7 (1.1)	3.8 (1.2)	3.7 (1.2)
Systolic blood pressure, Mean (SD)*†‡	128.5 (51.7)	134.4 (83.3)	125.0 (13.8)	138.1 (13.9)	122.2 (64.8)
Smoking status*†					
Non-smoker	26,893 (47.9%)	8,285 (42.4%)	18,608 (50.7%)	10,577 (49.4%)	16,316 (46.9%)
Ex-smoker	13,659 (24.3%)	6,143 (31.5%)	7,516 (20.5%)	5,841 (27.3%)	7,818 (22.5%)
Current smoker	7,829 (13.9%)	3,544 (18.1%)	4,285 (11.7%)	2,798 (13.1%)	5,031 (14.5%)
Unknown	7,816 (13.9%)	1,556 (8.0%)	6,260 (17.1%)	2,201 (10.3%)	5,615 (16.1%)
Alcohol consumption†					
No heavy drinking	33,730 (60.0%)	13,298 (68.1%)	20,432 (55.7%)	14,048 (65.6%)	19,682 (56.6%)
Heavy drinking	5,343 (9.5%)	2,013 (10.3%)	3,330 (9.1%)	2,168 (10.1%)	3,175 (9.1%)
Unknown	17,124 (30.5%)	4,217 (21.6%)	12,907 (35.2%)	5,201 (24.3%)	11,923 (34.3%)
Family history of CHD*	5,435 (9.7%)	2,174 (11.1%)	3,261 (8.9%)	1,966 (9.2%)	3,469 (10.0%)
Consultation frequency in prior 12 months, Median (IQR)	6 (3-11)	8 (4-14)	5 (2-10)	7 (4-13)	5 (2-10)
Medication use$					
Regular corticosteroids*	1,400 (2.5%)	976 (5.0%)	424 (1.2%)	660 (3.1%)	740 (2.1%)
Antihypertensives*	15,636 (27.8%)	8,018 (41.1%)	7,618 (20.8%)	9,590 (44.8%)	6,046 (17.4%)
Statins	8,875 (15.8%)	6,701 (34.3%)	2,174 (5.9%)	5,159 (24.1%)	3,716 (10.7%)
Antiplatelets	3,797 (6.8%)	2,377 (12.2%)	1,420 (3.9%)	2,068 (9.7%)	1,729 (5.0%)
Anticoagulants	1,430 (2.5%)	979 (5.0%)	451 (1.2%)	710 (3.3%)	720 (2.1%)
Comorbid condition					
Atrial fibrillation*	807 (1.4%)	767 (3.9%)	40 (0.1%)	447 (2.1%)	360 (1.0%)

Migraines*	2,830 (5.0%)	614 (3.1%)	2,216 (6.0%)	933 (4.4%)	1,897 (5.5%)
Diabetes*	5,626 (10.0%)	4,672 (23.9%)	954 (2.6%)	3,077 (14.4%)	2,549 (7.3%)
CKD stage 3-5*	4,931 (8.8%)	3,826 (19.6%)	1,105 (3.0%)	3,031 (14.2%)	1,900 (5.5%)
Chronic liver disease	962 (1.7%)	482 (2.5%)	480 (1.3%)	408 (1.9%)	554 (1.6%)
Chronic respiratory disease (not asthma)	4,191 (7.5%)	2,926 (15.0%)	1,265 (3.4%)	1,971 (9.2%)	2,220 (6.4%)
Asthma with recent OCS use$	4,585 (8.2%)	1,929 (9.9%)	2,656 (7.2%)	1,942 (9.1%)	2,643 (7.6%)
Asthma with no recent OCS use	7,435 (13.2%)	2,331 (11.9%)	5,104 (13.9%)	2,800 (13.1%)	4,635 (13.3%)
Severe mental illness / antipsychotic use*	1,101 (2.0%)	613 (3.1%)	488 (1.3%)	400 (1.9%)	701 (2.0%)
Dementia	1,121 (2.0%)	921 (4.7%)	200 (0.5%)	479 (2.2%)	642 (1.8%)
Chronic neurological disease	975 (1.7%)	523 (2.7%)	452 (1.2%)	413 (1.9%)	562 (1.6%)
Learning / intellectual disability	444 (0.8%)	190 (1.0%)	254 (0.7%)	139 (0.6%)	305 (0.9%)
Non-haematological cancer					
Diagnosed <1 year ago	1,951 (3.5%)	1,273 (6.5%)	678 (1.8%)	944 (4.4%)	1,007 (2.9%)
Diagnosed 1-4.9 years ago	2,044 (3.6%)	1,102 (5.6%)	942 (2.6%)	916 (4.3%)	1,128 (3.2%)
Diagnosed ≥5 years ago	3,610 (6.4%)	1,537 (7.9%)	2,073 (5.7%)	1,446 (6.8%)	2,164 (6.2%)
Haematological malignancy					
Diagnosed <1 year ago	261 (0.5%)	186 (1.0%)	75 (0.2%)	123 (0.6%)	138 (0.4%)
Diagnosed 1-4.9 years ago	161 (0.3%)	93 (0.5%)	68 (0.2%)	69 (0.3%)	92 (0.3%)
Diagnosed ≥5 years ago	137 (0.2%)	66 (0.3%)	71 (0.2%)	49 (0.2%)	88 (0.3%)
Rheumatoid arthritis*	831 (1.5%)	488 (2.5%)	343 (0.9%)	382 (1.8%)	449 (1.3%)
Systemic lupus erythematosus*	134 (0.2%)	67 (0.3%)	67 (0.2%)	54 (0.3%)	80 (0.2%)
HIV*	119 (0.2%)	35 (0.2%)	84 (0.2%)	50 (0.2%)	69 (0.2%)
Immunosuppression#	883 (1.6%)	471 (2.4%)	412 (1.1%)	389 (1.8%)	494 (1.4%)
Erectile dysfunction*	2,995 (13.2%)	2,270 (21.0%)	725 (6.1%)	1,522 (16.1%)	1,473 (11.2%)

*In QRISK3 algorithm, but non-imputed version included here (for smoking status, cholesterol:HDL ratio, systolic BP and BMI)
†most recent measure before baseline. N with missing cholesterol:HDL measurement 17,918 (31.9%)
‡Used on hypertension definition. N with missing systolic BP measurement 3,775 (6.7%)
$ at least 1 prescription in the 12 months before baseline. Other than corticosteroids which was defined as at least 2 prescriptions prior to baseline with the most recent ≤28 days before baseline
#ever history of solid organ transplant or permanent cellular immune deficiency; history in the 24 months before baseline for aplastic anaemia, bone marrow or stem cell transplant; history in the 12 months before baseline for biologics or other immunosuppressant therapy (excluding corticosteroids), other or unspecified cellular immune deficiency

Supplementary table 6. Hazard ratios for the effect of raised cardiovascular risk on severe outcomes after laboratory-confirmed SARS-CoV-2 from complete case analysis

	Crude N events	Complete case N events	Rate (95% CI) per 1,000 person-years	Crude HR (95% CI)	Crude HR (95% C.I.) in complete case analysis populations	Age- and sex-adjusted HR (95% CI)	Fully-adjusted# HR (95% CI)
	COVID-19 death*						
QRISK3 ≥10%	2,183	1,658	310.4 (297.7-323.7)	16.33 (14.61-18.24)	14.95 (13.07-17.10)	NA	8.77 (7.62-10.10)
QRISK3 <10%	365	244	20.4 (18.4-22.6)	1 (ref)	1 (ref)	NA	1 (ref)
Hypertension	1,384	786	156.2 (148.2-164.6)	2.27 (2.10-2.45)	2.08 (1.86-2.32)	1.08 (1.00-1.17)	1.05 (0.94-1.18)
No hypertension	1,164	504	72.6 (68.6-76.9)	1 (ref)	1 (ref)	1 (ref)	1 (ref)
	ICU admission†						
QRISK3 ≥10%	876	659	120.0 (112.1-128.6)	4.27 (3.83-4.76)	3.96 (3.47-4.51)	NA	3.66 (3.18-4.21)
QRISK3 <10%	573	375	29.9 (27.5-32.6)	1 (ref)	1 (ref)	NA	1 (ref)
Hypertension	768	430	82.2 (76.3-88.4)	2.16 (1.94-2.41)	1.90 (1.63-2.21)	1.55 (1.38-1.73)	1.15 (0.98-1.36)
No hypertension	681	302	40.2 (37.2-43.5)	1 (ref)	1 (ref)	1 (ref)	1 (ref)
	Respiratory support‡						
QRISK3 ≥10%	498	373	66.9 (61.1-73.3)	4.30 (3.72-4.98)	3.91 (3.28-4.66)	NA	3.73 (3.10-4.49)
QRISK3 <10%	320	210	16.6 (14.8-18.7)	1 (ref)	1 (ref)	NA	1 (ref)
Hypertension	452	245	47.9 (43.5-52.7)	2.39 (2.07-2.75)	1.97 (1.61-2.42)	1.73 (1.49-2.01)	1.20 (0.97-1.48)
No hypertension	366	168	21.3 (19.2-23.7)	1 (ref)	1 (ref)	1 (ref)	1 (ref)
	Hospitalisation$						
QRISK3 ≥10%	6,547	5,206	1212.4 (1183.4-1242.1)	4.41 (4.24-4.59)	4.39 (4.19-4.60)	NA	3.38 (3.22-3.56)
QRISK3 <10%	4,247	2,734	256.2 (248.6-264.0)	1 (ref)	1 (ref)	NA	1 (ref)
Hypertension	5,325	3,009	713.0 (694.1-732.4)	1.91 (1.84-1.99)	1.73 (1.64-1.82)	1.26 (1.21-1.31)	1.05 (0.99-1.11)
No hypertension	5,469	2,385	377.0 (367.1-387.1)	1 (ref)	1 (ref)	1 (ref)	1 (ref)
	Major adverse cardiovascular event						
QRISK3 ≥10%	570	449	82.4 (75.9-89.4)	7.51 (6.40-8.81)	6.95 (5.75-8.41)	NA	5.43 (4.44-6.64)
QRISK3 <10%	204	140	11.5 (10.0-13.1)	1 (ref)	1 (ref)	NA	1 (ref)
Hypertension	450	251	51.3 (46.8-56.3)	2.63 (2.28-3.04)	2.47 (2.00-3.04)	1.62 (1.40-1.87)	1.49 (1.20-1.85)
No hypertension	324	135	20.3 (18.2-22.6)	1 (ref)	1 (ref)	1 (ref)	1 (ref)

*Ascertained from ONS death certificate data in which the COVID related ICD-10 codes U07.1 or U07.2 were present in the record

[$]Ascertained from presence in CHESS dataset or HES APC record coded with primary diagnosis of U07.1 or U07.2
[†]Ascertained from CHESS records coded with ICU/HDU admission, only available for those with laboratory confirmed SARS-CoV-2
[‡]Ascertained from CHESS record coded with use of respiratory support via invasive mechanical ventilation, only available for those with laboratory confirmed SARS-CoV-2
[#]Hypertension models were adjusted for age, sex, ethnicity, socioeconomic status, body-mass index, alcohol consumption, smoking status, total cholesterol: high density lipoprotein cholesterol ratio, family history of coronary heart disease, treatment with corticosteroids, antiplatelets, or anticoagulants, diagnosis of atrial fibrillation, migraine, diabetes, chronic kidney disease stage 3-5, chronic liver disease, chronic lung disease, asthma, severe mental illness, dementia, chronic neurological disease, learning disability, or malignancy, and treatment or diagnosis of a immunosuppressive condition; and QRISK3 models were adjusted for alcohol consumption, treatment with antiplatelets or anticoagulants, diagnosis of chronic liver disease, chronic lung disease, asthma, dementia, chronic neurological disease, learning disability, or malignancy, and treatment or diagnosis of an immunosuppressive condition (which are not included in the QRISK3 algorithm).
Crude denominators: QRISK3 ≥10% = 39,295; QRISK3 <10% = 107,465; hypertension = 49,955; no hypertension = 96,805. Complete case denominators: QRISK3 ≥10% = 30,506; QRISK3 <10% = 67,219; hypertension = 24,362; no hypertension = 32,902.

Supplementary table 7. Baseline characteristics of those included in and excluded from complete case analysis for the laboratory-confirmed SARS-CoV-2 study population

	QRISK3 score				Hypertension			
	**Raised risk included**	**Raised risk excluded**	**Low risk included**	**Low risk excluded**	**Raised risk included**	**Raised risk excluded**	**Low risk included**	**Low risk excluded**
	N=30,506	N=8,789	N=67,219	N=40,246	N=36,844	N=13,111	N=60,881	N=35,924
Age (years), Mean (SD)*	65.2 (9.5)	65.7 (9.3)	50.4 (6.6)	49.2 (6.5)	58.4 (10.6)	55.6 (10.3)	52.9 (10.1)	50.9 (8.9)
Age group (years)*								
40-54	4,174 (13.7%)	976 (11.1%)	48,589 (72.3%)	31,189 (77.5%)	14,684 (39.9%)	6,698 (51.1%)	38,079 (62.6%)	25,467 (70.9%)
55-64	10,797 (35.4%)	3,170 (36.1%)	17,291 (25.7%)	8,499 (21.1%)	12,359 (33.5%)	4,076 (31.1%)	15,729 (25.8%)	7,593 (21.1%)
65-74	9,952 (32.6%)	2,933 (33.4%)	1,339 (2.0%)	558 (1.4%)	6,318 (17.2%)	1,501 (11.4%)	4,973 (8.2%)	1,990 (5.6%)
75-84	5,583 (18.3%)	1,710 (19.5%)	0 (0.0%)	0 (0.0%)	3,483 (9.4%)	836 (6.4%)	2,100 (32.4%)	874 (2.4%)
Sex*†								
Women	11,294 (37.0%)	3,022 (34.4%)	43,440 (64.6%)	23,049 (57.3%)	18,408 (50.0%)	6,200 (47.3%)	36,326 (59.7%)	19,871 (55.3%)
Men	19,212 (63.0%)	5,767 (65.6%)	23,779 (35.4%)	17,197 (43.7%)	18,436 (50.0%)	6,911 (52.7%)	24,555 (40.3%)	16,053 (44.7%)
Comorbid condition								
Atrial fibrillation*	1,030 (3.4%)	273 (3.1%)	68 (0.1%)	49 (0.1%)	605 (1.6%)	140 (1.1%)	493 (0.8%)	182 (0.5%)
Diabetes*	8,644 (28.3%)	1,167 (13.3%)	2,031 (3.0%)	396 (1.0%)	5,879 (16.0%)	724 (5.5%)	4,796 (7.9%)	839 (2.3%)
CKD stage 3-5*	5,746 (18.8%)	1,697 (2.5%)	1,267 (14.4%)	584 (1.5%)	4,546 (12.3%)	936 (7.1%)	2,897 (4.8%)	915 (2.6%)
Chronic liver disease	671 (2.2%)	104 (1.2%)	594 (0.9%)	194 (0.5%)	538 (1.5%)	103 (0.8%)	727 (1.2%)	195 (0.5%)
Chronic respiratory disease	2,722 (8.9%)	581 (6.6%)	1,189 (1.8%)	388 (1.0%)	1,884 (5.1%)	383 (2.9%)	2,027 (3.3%)	586 (1.6%)
Asthma - recent OCS use$	2,128 (7.0%)	469 (5.3%)	3,509 (5.2%)	1,452 (3.6%)	2,488 (6.8%)	632 (4.2%)	3,149 (5.2%)	1,289 (3.6%)
Asthma - no recent OCS use	2,874 (9.4%)	708 (8.1%)	7,355 (10.9%)	3,924 (9.8%)	3,804 (10.3%)	1,252 (9.6%)	6,425 (10.6%)	3,380 (9.4%)

*In QRISK3 algorithm, but non-imputed version included here
†3 people were unknown sex were included in the study population, but those with recorded sex are shown here
$ at least 1 prescription in the 12 months before baseline

Supplementary table 8. The number and proportion of patients with outcomes of interest by age group and QRISK3 score level

Age group	QRISK3 score	All study population	COVID-19 death	ICU	Respiratory support	Hospitalisation	MACE
40-54	<10%	79,778 (93.9%)	170 (74.6%)	346 (78.5%)	196 (76.6%)	2,750 (81.2%)	119 (73.5%)
	≥10%	5,150 (6.1%)	58 (25.4%)	95 (21.5%)	60 (23.4%)	556 (16.8%)	43 (26.5%)
55-64	<10%	25,790 (64.9%)	163 (36.0%)	205 (40.8%)	111 (39.2%)	1,352 (45.9%)	75 (34.1%)
	≥10%	13,967 (35.1%)	290 (64.0%)	298 (59.2%)	172 (60.8%)	1,593 (54.1%)	145 (65.9%)
65-74	<10%	1,897 (12.8%)	32 (4.2%)	22 (5.9%)	13 (5.9%)	145 (6.0%)	10 (4.8%)
	≥10%	12,885 (87.2%)	737 (95.8%)	353 (94.1%)	206 (94.1%)	2,255 (94.0%)	199 (95.2%)
75-84	<10%	0 (0.0%)	0 (0.0%)	0 (0.0%)	0 (0.0%)	0 (0.0%)	0 (0.0%)
	≥10%	7,293 (100.0%)	1,098 (100.0%)	130 (100.0%)	60 (100.0%)	2,143 (100.0%)	183 (100.0%)

Supplementary table 9. Hazard ratios for the effect of raised QRISK3 score on severe outcomes after laboratory-confirmed SARS-CoV-2 with age-adjustment and age stratification from complete case analysis

	Crude HR (95% CI)	Adjusted[#] HR (95% CI)	Further age-adjusted HR (95% CI)	Adjusted[#] HR (95% CI) ages 40-54 years	Adjusted[#] HR (95% CI) ages 55-64 years	Adjusted[#] HR (95% CI) ages 65-74 years
COVID-19 death*						
QRISK3 ≥10%	15.82 (14.17-17.67)	8.77 (7.62-10.10)	2.91 (2.45-3.45)	4.32 (3.05-6.12)	2.72 (2.15-3.43)	2.90 (1.87-4.50)
QRISK3 <10%	1 (ref)	1 (ref)	1 (ref)	1 (ref)	1 (ref)	1 (ref)
ICU admission[†]						
QRISK3 ≥10%	3.99 (3.58-4.45)	3.66 (3.18-4.21)	3.10 (2.60-3.69)	3.98 (3.01-5.26)	2.33 (1.88-2.90)	2.31 (1.34-3.96)
QRISK3 <10%	1 (ref)	1 (ref)	1 (ref)	1 (ref)	1 (ref)	1 (ref)
Respiratory support[‡]						
QRISK3 ≥10%	4.07 (3.52-4.71)	3.73 (3.10-4.49)	3.15 (2.50-3.97)	4.01 (2.78-5.79)	2.39 (1.79-3.21)	2.75 (1.29-5.90)
QRISK3 <10%	1 (ref)	1 (ref)	1 (ref)	1 (ref)	1 (ref)	1 (ref)
Hospitalisation[$]						
QRISK3 ≥10%	4.27 (4.11-4.44)	3.38 (3.22-3.56)	2.24 (2.10-2.38)	2.88 (2.59-3.20)	2.18 (2.00-2.37)	2.27 (1.85-2.79)
QRISK3 <10%	1 (ref)	1 (ref)	1 (ref)	1 (ref)	1 (ref)	1 (ref)
Major adverse cardiovascular event						
QRISK3 ≥10%	7.05 (6.02-8.26)	5.43 (4.44-6.64)	3.38 (2.64-4.32)	4.45 (2.97-6.66)	3.26 (2.34-4.53)	3.23 (1.42-7.32)
QRISK3 <10%	1 (ref)	1 (ref)	1 (ref)	1 (ref)	1 (ref)	1 (ref)

*Ascertained from ONS death certificate data in which the COVID related ICD-10 codes U07.1 or U07.2 were present in the record
[$]Ascertained from presence in CHESS dataset or HES APC record coded with primary diagnosis of U07.1 or U07.2
[†]Ascertained from CHESS records coded with ICU/HDU admission, only available for those with laboratory confirmed SARS-CoV-2
[‡]Ascertained from CHESS record coded with use of respiratory support via invasive mechanical ventilation, only available for those with laboratory confirmed SARS-CoV-2
[#]Adjusted for alcohol consumption, treatment with antiplatelets or anticoagulants, diagnosis of chronic liver disease, chronic lung disease, asthma, dementia, chronic neurological disease, learning disability, or malignancy, and treatment or diagnosis of an immunosuppressive condition (which are not included in the QRISK3 algorithm).

Supplementary table 10. Hazard ratios for the effect of raised cardiovascular risk on severe outcomes after laboratory-confirmed SARS-CoV-2 from complete case analysis stratified by wave of the pandemic

	Wave 1				Wave 2			
	N events	Rate (95% CI) per 1,000 person-years	Crude HR (95% CI)	Fully-adjusted# HR (95% CI)	N events	Rate (95% CI) per 1,000 person-years	Crude HR (95% CI)	Fully-adjusted# HR (95% CI)
COVID-19 death*								
QRISK3 ≥10%	1249	356.0 (336.8-376.3)	11.10 (9.61-12.83)	7.04 (5.84-8.50)	934	265.1 (248.6-282.6)	18.28 (15.39-21.72)	9.32 (7.52-11.55)
QRISK3 <10%	215	28.3 (24.7-32.3)	1 (ref)	1 (ref)	150	14.6 (12.5-17.2)	1 (ref)	1 (ref)
Hypertension	774	185.2 (172.6-198.8)	1.81 (1.64-2.01)	1.05 (0.90-1.22)	610	130.3 (120.3-141.0)	2.53 (2.24-2.85)	1.14 (0.95-1.36)
No hypertension	690	99.6 (92.4-107.3)	1 (ref)	1 (ref)	474	52.1 (47.6-57.0)	1 (ref)	1 (ref)
ICU admission†								
QRISK3 ≥10%	561	154.3 (141.5-168.3)	2.74 (2.38-3.14)	2.88 (2.41-3.44)	315	87.5 (78.3-97.9)	4.75 (3.96-5.69)	4.02 (3.20-5.05)
QRISK3 <10%	374	45.5 (40.9-50.6)	1 (ref)	1 (ref)	199	18.7 (16.2-21.5)	1 (ref)	1 (ref)
Hypertension	505	114.5 (104.4-125.5)	1.86 (1.62-2.12)	1.24 (1.01-1.53)	263	54.5 (48.2-61.7)	2.08 (1.75-2.48)	1.14 (0.88-1.46)
No hypertension	430	58.3 (52.8-64.3)	1 (ref)	1 (ref)	251	26.8 (23.6-30.4)	1 (ref)	1 (ref)
Respiratory support‡								
QRISK3 ≥10%	374	100.0 (89.9-111.2)	2.63 (2.22-3.11)	2.83 (2.28-3.51)	124	35.0 (29.3-41.8)	5.59 (4.14-7.55)	4.81 (3.32-6.98)
QRISK3 <10%	255	30.8 (27.0-35.0)	1 (ref)	1 (ref)	65	6.3 (5.0-8.1)	1 (ref)	1 (ref)
Hypertension	349	78.0 (69.9-87.1)	1.99 (1.68-2.35)	1.31 (1.02-1.68)	103	21.8 (18.0-26.5)	2.36 (1.77-3.14)	1.08 (0.72-1.60)
No hypertension	280	37.1 (32.8-42.0)	1 (ref)	1 (ref)	86	9.5 (7.7-11.7)	1 (ref)	1 (ref)
Hospitalisation$								
QRISK3 ≥10%	2870	1299.2 (1252.5-1347.6)	3.10 (2.92-3.29)	2.83 (2.61-3.06)	3677	1152.3 (1115.6-1190.1)	4.41 (4.19-4.64)	3.36 (3.15-3.58)
QRISK3 <10%	1687	257.1 (245.2-269.7)	1 (ref)	1 (ref)	2560	255.6 (245.9-265.7)	1 (ref)	1 (ref)
Hypertension	2261	737.0 (707.3-768.0)	1.57 (1.48-1.67)	1.08 (0.99-1.18)	3064	696.3 (672.0-721.4)	1.96 (1.86-2.05)	1.07 (1.00-1.16)
No hypertension	2296	402.7 (386.5-419.5)	1 (ref)	1 (ref)	3173	360.4 (348.0-373.1)	1 (ref)	1 (ref)
Major adverse cardiovascular event								
QRISK3 ≥10%	304	89.1 (79.6-99.7)	5.74 (4.58-7.18)	5.04 (3.77-6.74)	266	75.9 (67.3-85.6)	7.69 (6.12-9.66)	5.21 (3.93-6.90)
QRISK3 <10%	102	13.5 (11.1-16.4)	1 (ref)	1 (ref)	102	10.0 (8.2-12.1)	1 (ref)	1 (ref)
Hypertension	239	58.3 (51.4-66.2)	2.31 (1.90-2.82)	1.62 (1.20-2.19)	211	45.2 (39.5-51.7)	2.66 (2.16-3.27)	1.42 (1.03-1.95)
No hypertension	167	24.3 (20.9-28.2)	1 (ref)	1 (ref)	157	17.3 (14.8-20.2)	1 (ref)	1 (ref)

*Ascertained from ONS death certificate data in which the COVID related ICD-10 codes U07.1 or U07.2 were present in the record
$Ascertained from presence in CHESS dataset or HES APC record coded with primary diagnosis of U07.1 or U07.2
†Ascertained from CHESS records coded with ICU/HDU admission, only available for those with laboratory confirmed SARS-CoV-2

[‡]Ascertained from CHESS record coded with use of respiratory support via invasive mechanical ventilation, only available for those with laboratory confirmed SARS-CoV-2

[#]Hypertension models were adjusted for age, sex, ethnicity, socioeconomic status, body-mass index, alcohol consumption, smoking status, total cholesterol: high density lipoprotein cholesterol ratio, family history of coronary heart disease, treatment with corticosteroids, antiplatelets, or anticoagulants, diagnosis of atrial fibrillation, migraine, diabetes, chronic kidney disease stage 3-5, chronic liver disease, chronic lung disease, asthma, severe mental illness, dementia, chronic neurological disease, learning disability, or malignancy, and treatment or diagnosis of a immunosuppressive condition; and QRISK3 models were adjusted for alcohol consumption, treatment with antiplatelets or anticoagulants, diagnosis of chronic liver disease, chronic lung disease, asthma, dementia, chronic neurological disease, learning disability, or malignancy, and treatment or diagnosis of an immunosuppressive condition (which are not included in the QRISK3 algorithm).

Supplementary table 11. Hazard ratios for the effect of raised cardiovascular risk on severe outcomes after clinically reported COVID-19 from complete case analysis

	N events	Rate (95% CI) per 1,000 person-years	Crude HR (95% CI)	Age- and sex-adjusted HR (95% CI)	Fully-adjusted# HR (95% CI)
COVID-19 death*					
QRISK3 ≥10%	286	34.6 (30.8-38.8)	13.82 (9.89-19.31)	NA	7.39 (4.83-11.31)
QRISK3 <10%	39	2.5 (1.8-3.5)	1 (ref)	NA	1 (ref)
Hypertension	162	17.6 (15.1-20.6)	1.61 (1.29-2.00)	0.82 (0.66-1.03)	1.07 (0.75-1.53)
No hypertension	163	11.2 (9.6-13.1)	1 (ref)	1 (ref)	1 (ref)
Hospitalisation$					
QRISK3 ≥10%	1,312	168.7 (159.8-178.1)	2.57 (2.36-2.79)	NA	2.05 (1.84-2.28)
QRISK3 <10%	965	64.4 (60.5-68.6)	1 (ref)	NA	1 (ref)
Hypertension	1,087	124.3 (117.2-132.0)	1.49 (1.37-1.61)	1.16 (1.07-1.26)	1.00 (0.88-1.13)
No hypertension	1,190	84.9 (80.2-89.8)	1 (ref)	1 (ref)	1 (ref)
Major adverse cardiovascular event					
QRISK3 ≥10%	531	65.4 (60.1-71.2)	6.81 (5.67-8.18)	NA	5.09 (4.06-6.37)
QRISK3 <10%	147	9.6 (8.1-11.2)	1 (ref)	NA	1 (ref)
Hypertension	400	44.1 (40.0-48.7)	2.32 (1.99-2.70)	1.50 (1.28-1.75)	1.26 (1.00-1.58)
No hypertension	278	19.3 (17.1-21.7)	1 (ref)	1 (ref)	1 (ref)

*Ascertained from ONS death certificate data in which the COVID related ICD-10 codes U07.1 or U07.2 were present in the record

$Ascertained from presence in CHESS dataset or HES APC record coded with primary diagnosis of U07.1 or U07.2

#Hypertension models were adjusted for age, sex, ethnicity, socioeconomic status, body-mass index, alcohol consumption, smoking status, total cholesterol: high density lipoprotein cholesterol ratio, family history of coronary heart disease, treatment with corticosteroids, antiplatelets, or anticoagulants, diagnosis of atrial fibrillation, migraine, diabetes, chronic kidney disease stage 3-5, chronic liver disease, chronic lung disease, asthma, severe mental illness, dementia, chronic neurological disease, learning disability, or malignancy, and treatment or diagnosis of a immunosuppressive condition; and QRISK3 models were adjusted for alcohol consumption, treatment with antiplatelets or anticoagulants, diagnosis of chronic liver disease, chronic lung disease, asthma, dementia, chronic neurological disease, learning disability, or malignancy, and treatment or diagnosis of an immunosuppressive condition (which are not included in the QRISK3 algorithm).

Supplementary table 12. Hazard ratios for the effect of raised cardiovascular risk with refined QRISK3 score categories on severe outcomes after laboratory-confirmed SARS-CoV-2 from complete case analysis

	N events	Rate (95% CI) per 1,000 person-years	Crude HR (95% CI)	Fully-adjusted# HR (95% CI)
COVID-19 death*				
QRISK3 ≥20%	1,589	561.7 (534.7-590.0)	30.81 (27.49-34.52)	15.15 (13.05-17.59)
QRISK3 10-<20%	594	141.3 (130.4-153.2)	7.23 (6.35-8.24)	5.32 (4.54-6.23)
QRISK3 <10%	365	20.4 (18.4-22.6)	1 (ref)	1 (ref)
ICU admission†				
QRISK3 ≥20%	411	137.2 (124.0-151.8)	5.07 (4.45-5.79)	4.21 (3.54-5.02)
QRISK3 10-<20%	465	108.5 (98.8-119.1)	3.76 (3.31-4.27)	3.40 (2.91-3.96)
QRISK3 <10%	573	29.9 (27.5-32.6)	1 (ref)	1 (ref)
Respiratory support‡				
QRISK3 ≥20%	230	75.9 (66.3-86.8)	5.06 (4.24-6.04)	4.63 (3.68-5.83)
QRISK3 10-<20%	268	60.9 (53.8-68.9)	3.82 (3.23-4.52)	3.31 (2.70-4.07)
QRISK3 <10%	320	16.6 (14.8-18.7)	1 (ref)	1 (ref)
Hospitalisation$				
QRISK3 ≥20%	3,769	1903.1 (1843.3-1964.8)	6.73 (6.44-7.04)	4.77 (4.50-5.07)
QRISK3 10-<20%	2,778	812.4 (782.7-843.1)	3.01 (2.87-3.16)	2.70 (2.55-2.86)
QRISK3 <10%	4,247	256.2 (248.6-264.0)	ref	ref
Major adverse cardiovascular event				
QRISK3 ≥20%	343	124.2 (111.7-138.0)	11.54 (9.71-13.73)	8.11 (6.46-10.17)
QRISK3 10-<20%	227	54.6 (48.0-62.2)	4.92 (4.07-5.94)	4.10 (3.27-5.14)
QRISK3 <10%	204	11.5 (10.0-13.1)	ref	ref

*Ascertained from ONS death certificate data in which the COVID related ICD-10 codes U07.1 or U07.2 were present in the record
$Ascertained from presence in CHESS dataset or HES APC record coded with primary diagnosis of U07.1 or U07.2
†Ascertained from CHESS records coded with ICU/HDU admission, only available for those with laboratory confirmed SARS-CoV-2
‡Ascertained from CHESS record coded with use of respiratory support via invasive mechanical ventilation, only available for those with laboratory confirmed SARS-CoV-2
#Adjusted for alcohol consumption, treatment with antiplatelets or anticoagulants, diagnosis of chronic liver disease, chronic lung disease, asthma, dementia, chronic neurological disease, learning disability, or malignancy, and treatment or diagnosis of an immunosuppressive condition (which are not included in the QRISK3 algorithm).

Supplementary figure 1. Adjusted hazard ratios in hypertension model for the association between laboratory-confirmed SARS-CoV-2 and COVID-19 death from complete case analysis

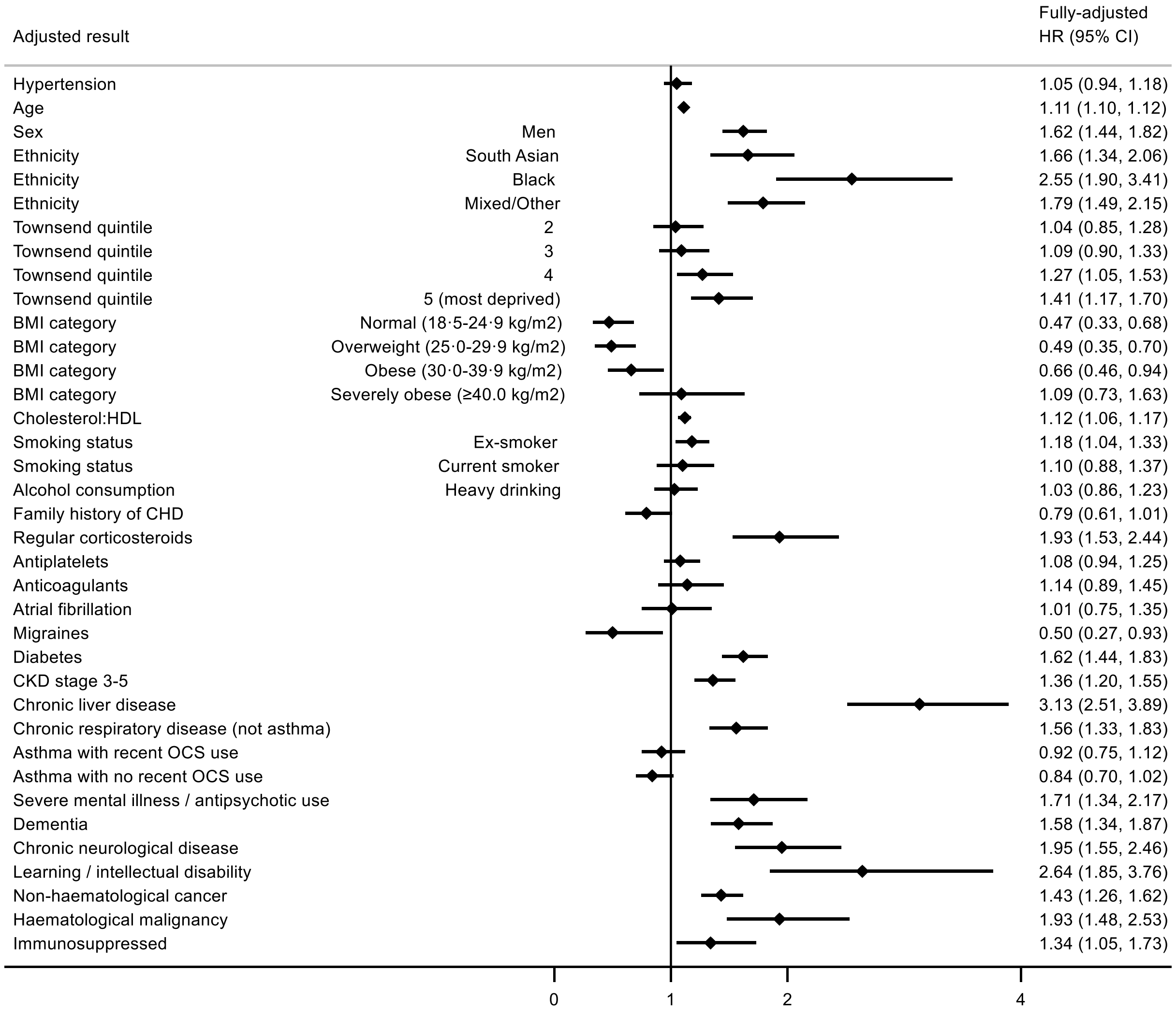

Supplement: Supplementary Methods S1 and S2, Tables S1–S12, and Fig. S1 [file mmc1.pdf]
